# Causal language jumps and non-alignments between clinical practice guidelines and original studies: a systematic evaluation of diabetes guidelines and their cited evidence

**DOI:** 10.1136/bmjopen-2025-109205

**Published:** 2026-02-05

**Authors:** Keling Wang, Chang Wei, Jeremy A Labrecque

**Affiliations:** 1 Department of Epidemiology, Erasmus MC, Rotterdam, The Netherlands

**Keywords:** Protocols & guidelines, GENERAL MEDICINE (see Internal Medicine), DIABETES & ENDOCRINOLOGY, STATISTICS & RESEARCH METHODS

## Abstract

**Abstract:**

**Objectives:**

Clinical practice guidelines are designed to guide clinical practice and often make causal claims when making recommendations. Sometimes, guidelines make or require stronger causal claims than supplied in the original studies, a phenomenon we call ‘causal language jump’. We aimed to evaluate the strength of expressed causation in guidelines and the evidence they reference to assess the pattern of jumps, taking diabetes as an illustrative example.

**Design:**

This is a systematic evaluation of guidelines and original studies cited by them, using scoping review design with deviations.

**Data source:**

Randomly sampled 300 guideline statements (narrative sentences describing evidence to support recommendations) from four selected diabetes guidelines.

**Eligibility criteria:**

The eligible guidelines should focus on non-pharmacological treatments or preventive strategies for adult type 2 diabetes mellitus management and related complications. The eligible action recommendations and guideline statements should intend to support non-pharmacological treatments or preventive strategies of type 2 diabetes or in a general diabetic context.

**Data extraction and synthesis:**

We rated the causation strength in the statements and the dependence on causation in recommendations supported by these statements using existing scales. Among the causal statements, the cited original studies were similarly assessed. We then evaluated jumps by checking if the causal claims in guideline statements were stronger than in original studies, and if the causation-dependence in guideline recommendations was stronger than supplied in guideline statements. We also assessed how well they report target trial emulation (TTE) components as a proxy for reliability.

**Results:**

Of the 300 statements, 114 (38.0%) were causal, and 76 (66.7%) expressed strong causation. 27.2% (31/114) of causal guideline statements stated stronger causation than any of their references and demonstrated ‘causal language jump’; 34.9% (29/83) of guideline recommendations required stronger causation than provided in statements. Of the 53 eligible studies for TTE rating, most did not report treatment assignment and causal contrast in detail. The prevalence of these jumps could be partially attributed to the suboptimal use of causal and associational words.

**Conclusions:**

Causal language jumps were common among diabetes guidelines. While these jumps are sometimes inevitable, they should always be justified by good causal inference practices.

**STRENGTHS AND LIMITATIONS OF THIS STUDY**

- We introduced the concepts of ‘causation flow’ and ‘causal language jump’ to describe non-alignment between guidelines and original studies.
- By assessing jumps along the whole ‘flow’ of causation from original studies to guidelines, we did not only investigate the causal language use in articles or in guidelines, but also the relationship between the two.
- We assessed the alignment of causation strength in guidelines and in the original studies they cited using an existing well-performed scale of the strength of causal language.
- We used a target trial emulation-based strategy to see how well the necessary components are reported to support their causal claims.
- Several causal evidence pieces, each with weaker strength, could be synthesised into stronger evidence to sufficiently support a causal claim or recommendation, but we cannot look into the characteristics of such ‘joint support’ at semantic level.

## Introduction

 Clinical practice guidelines (abbreviated as ‘guidelines’) are ‘(A) set of statements that include recommendations intended to optimise patient care that are informed by a systematic review of evidence and an assessment of the benefits and harms of alternative care options’.^1^ Guidelines have become increasingly important in clinical decision-making since evidence-based care has gained attention,^2^ and thus recommendations from guidelines often heavily impact actual treatment decisions or care strategies.

Guidelines often concern an action or treatment decision. Guideline recommendations that aim to inform practitioners about giving or withholding treatment are necessarily causal in nature, because they compare the consequences of two (or more) potential treatment options. Causation, however, is difficult to demonstrate, and causal evidence often requires very careful interpretation. If the causal statements in guidelines are not aligned with the evidence on which it relies, practitioners may make suboptimal decisions affecting patient or population health.

**Figure 1:**
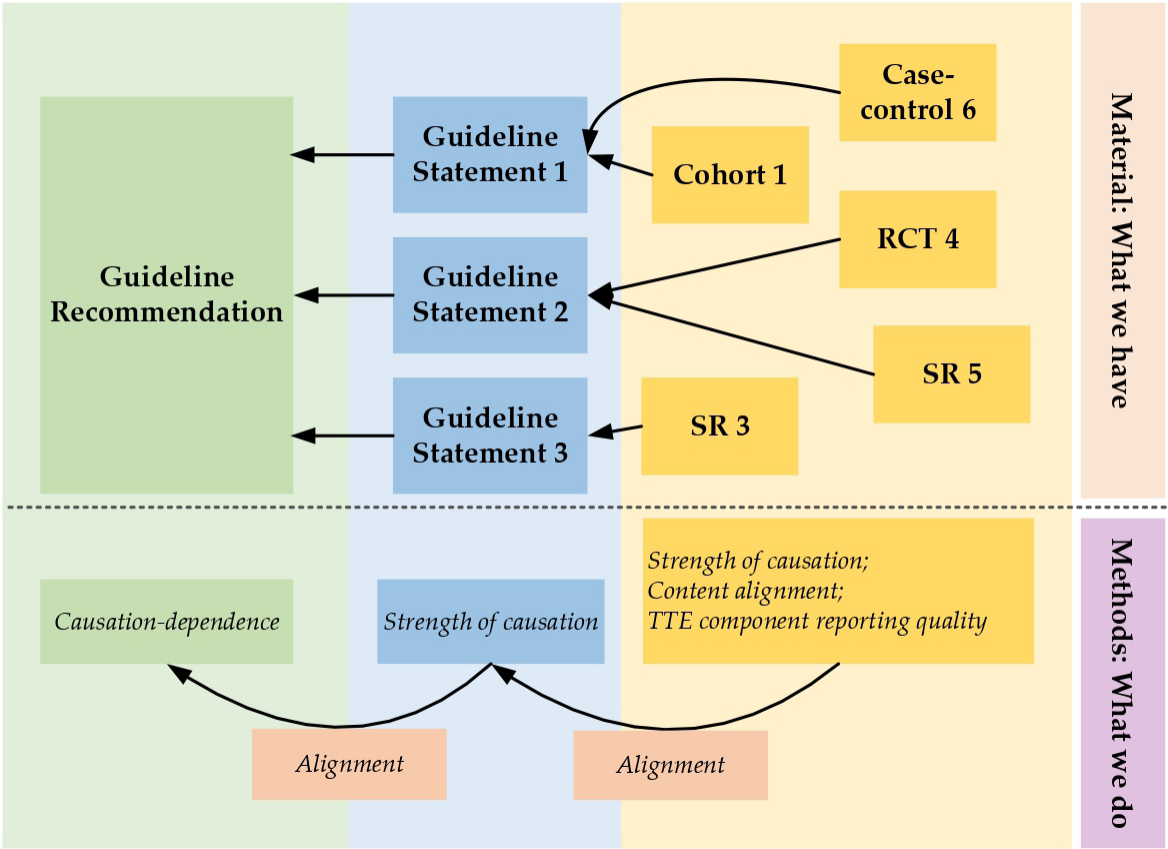
Structure of causation flows in a clinical practice guideline and the assessment of causal language jumps along this flow. The upper panel shows the basic structure of a guideline as well as its original studies; the lower panel gives an overview of our methods for the assessment of causal language jumps and misalignment along the entire causation flow. RCT, randomised controlled trial; SR, systematic review; TTE, target trial emulation.

The strength of causation expressed in guidelines should be adequately supported by the existing evidence they reference. The strength of causal conclusions should flow from the original studies to the statements in guidelines that narrate evidence, and finally to the guideline recommendations that inform clinical practice (figure 1). To ensure the validity of a recommendation, all causal information should be faithfully passed in this flow.

However, the flow of the strength of causal language may not always be well-supported. The strength of causation expressed in guidelines may be stronger than that expressed in the literature. For example, observational studies often use associational language^3 4^ but are still used to support causal statements in guidelines. There could also be recommendations that rely on a larger assumption set than the ones implied in their corresponding original studies.

We use the term ‘causal language jump’ to refer to the situations where the causation or the dependence to causation is stronger than the previous step in the causation flow. In a guideline’s causation flow, a causal language jump could be non-alignment of the expressed strength of causation or a gap from non-causal to causal statements when passing evidence from original studies to guideline statements and finally recommendations. Attention should be paid to this discontinuity as they can have consequences in real-world clinical decision-making. Whether and how causal language jumps are made in guidelines, however, is not known yet.

Causal language in scientific literature often consists of a set of words, modifiers and specific sentence structures to express the existence, confidence, uncertainty, magnitude and other attributes of causality,^5^,^7^ which is fundamental to modern epidemiology and clinical studies.^8^ The causal linking words play a central role in causal language, and they express whether a (counterfactual) change exists and the direction of this change. Examples of such words include ‘effect’, ‘benefit’, ‘impact’ and ‘reduce’.^3 7^ There have been studies assessing the use of causal language,^79^,^12^ finding inconsistency in expressions of causation. For example, Haber *et al*^7^ rated the causation strength expressed in abstracts and main texts of more than 1000 articles and suggested the existence of a disconnect between the use of causal language and the implications in these articles. There are also published studies in the field of health communication,^13^ media^12 14^ and computer science^15^ that note this disconnect between the intention and expression of causation.

These studies on causal language use, however, focus on observational studies themselves, while little work has been done on how causal or non-causal language can have a larger impact, for example, on guidelines. In other words, they did not assess the full ‘causation flow’, but only the first stop on this flow. To get a full picture of causal language use and potential jumps in guidelines, we need to assess causal language not only within the guidelines or original studies themselves, but also between them.

Therefore, we aimed to evaluate the use of causal language in guidelines and the original studies they cited, and illustrate the pattern of potential causal language jumps by checking the alignment of causal language. We focused on clinical guidelines that provided recommendations for non-pharmacological prevention, treatment and management of adults with type 2 diabetes mellitus (T2DM), because the evidence for non-pharmacological treatments is well-developed for T2DM^16^ but is also more vulnerable to biases and can potentially provide a good illustration for the issue we examined.

## Methods

### Study design

We took the Joanna Briggs Institute Scoping Review Guideline^17^ as reference to design our workflow but with deviations, as we did not perform a standard scoping review. The protocol of this study was registered at the Open Science Framework.^18^ The reporting of this study followed the Preferred Reporting Items for Systematic Reviews and Meta-Analyses extension for Scoping Reviews^19^ guideline. The workflow is displayed in figure 1 and is described in detail below. Briefly, (1) we selected eligible guideline recommendations and their surrounding guideline statements; (2) we randomly sampled from these guideline statements, included the recommendations they support, and assessed the strength of causal language in statements or the dependence to causal evidence in recommendations; (3) we collected the original studies these statements cited, assessed the strength of causal language and assessed the alignment between the two and (4) we checked the reporting quality of the eligible original studies in terms of target trial emulation (TTE) components^20^ to see whether the quality sufficed to support their conclusions.

### Guideline selection

The eligibility criteria for guidelines are: (1) they should focus on non-pharmacological treatments or preventive strategies; (2) they should be developed for adult T2DM management and related complications and (3) they should have clear quality of evidence ratings, strength of each recommendation item, development methodologies, and should be the most updated version written in English. We selected diabetes guidelines together with diabetologists and expert researchers in the field of diabetes from the eligible ones that were developed by internationally recognised academic associations. Eight chapters from four sets of guidelines^21^,^28^ were selected according to both the predefined criteria and expert suggestions.

### Recommendation and guideline statement selection

All action recommendations (figure 1, leftmost column) from the guideline sections mentioned above were eligible for assessment if they focused on non-pharmacological treatment or preventive strategies of T2DM or in a general diabetic context. We manually picked all these eligible recommendations for further analysis.

These recommendations were typically surrounded and supported by a number of sentences that were used to narrate evidence and provide support to recommendations, which we referred to as guideline statements (figure 1, middle column). All the guideline statements attached to an eligible action recommendation were also considered eligible as long as they focused on non-pharmacological treatment strategies. Due to our capacity limit, we randomly drew 300 statements and picked their associated action recommendations from all eligible ones to proceed. The random sampling of these statements was done in R V.4.4 using function ‘base::sample’ with a fixed seed number 114514.

### Recommendation and guideline statement assessment

We rated each of the 300 statements for the expressed strength of causation based on causal linking words, modifiers and sentence tone about uncertainty. The causal linking words were central to our rating, and we took a list describing causal implications of different causal linking words from a previous study.^7^ For the associated recommendation, we rated causation-dependence, that is, to what extent the recommendation relied on causal evidence. This was judged on routine clinical practice and subject-matter knowledge. Subsequently, we checked whether one recommendation can be effectively supported in terms of causation strength, by comparing its causation-dependence and all the causation strength ratings of its surrounding guideline statements.

A four-level scale, namely The Causal Implication Strength Rating Scale,^7^ was used for causation and causation-dependence ratings. We rated each statement as ineligible (i) if they were related to pharmacological treatments, no relationship expressed between variables (−1), correlational but not causal (0), weak causal (1), moderate causal (2) and strong/explicit causal (3); each recommendation as no causal evidence needed to make recommendations (0), little causal evidence needed (1), some or possible causal evidence needed (2) and definitely explicit causal evidence needed (3). Detailed explanations and examples of these ratings can be found in online supplemental file S1.

### Original study selection and assessment

For each guideline statement that was sampled and rated as a causal one, we extracted all eligible studies cited by this statement (figure 1, rightmost column). Eligible cited studies were intervention studies, observational studies, systematic reviews of intervention and/or observational studies, or aggregated evidence. If there was no study cited, we checked surrounding sentences to determine whether they contained a reference that might support the statement.

For each original study, we extracted one conclusive sentence from the main text that best supported the associated guideline statement. These sentences were rated for their strength of causation using the same scale described above. Additionally, a three-level item was used to rate for the alignment of the contents of these potentially causal sentences to those in the guideline statements (‘not aligned’, ‘partially aligned’ and ‘completely aligned’). This alignment score was used to assess whether original studies could effectively support guideline statements in terms of contents. Examples of content alignment ratings can be found in online supplemental file S1.

After assessing causation strength and alignment in the main text, we randomly picked one original study for each guideline statement (if there existed more than one) to further assess the reporting quality. The random draw of such study was done in R V.4.4 using function ‘base::sample()’ with a fixed seed number 114514.

Further assessment was done in two additional steps. First, we extracted one conclusive sentence and one action recommendation sentence in the abstract. These abstract sentences were rated similarly to those in the main texts, using the same scale. By this, we aimed to verify whether the conclusive sentences in abstracts carried causation strength of similar patterns and alignment compared with those from main texts.

Second, to assess how well the design and study details were reported to support a causal claim in the original studies as a proxy for the reliability and quality of the claim, we extracted the study components or corresponding emulated trial components for primary reports (single trials and observational studies) among these selected studies. We evaluated them in terms of assessing the reporting of (target) trial components under a TTE Framework.^20 29^ This included checking the reporting of eligibility criteria, treatment strategies, assignment procedures, follow-up period, outcome, causal contrast and analysis plan; each of them was rated as 0 (not or inappropriately reported), 1 (partially or problematically reported) or 2 (fully reported).^30^ Explanations of the TTE component reporting quality scale can be found in online supplemental file S1.

### Divergence resolving

Ratings were done by two coauthors (KW and JAL for guideline ratings; KW and CW for original study ratings) independently. The ratings were then compared and assessed for agreement. All the discrepancies in guideline statement ratings and guideline recommendation ratings were discussed and resolved by KW, CW and JAL together. All other discrepancies in original study sentences and reporting quality ratings were discussed and resolved by two coauthors together. Data extraction table for study characteristics and other data selection and extraction components was done by one author (KW) and checked by the second (CW).

### Data synthesis and statistical considerations

A narrative and descriptive summary is presented. For categorical variables, the count and proportion are reported. For continuous variables, means and SDs, or medians and IQR are reported accordingly. All statistical analyses were implemented using R V.4.4 in RStudio.

### Patient and public involvement

None.

## Results

### Causal language use and alignment within guidelines

The guideline characteristics are displayed in table 1, and the rationale for including them was outlined in online supplemental file S2. From these guidelines, we extracted 1175 guideline statements and 183 action recommendations. Of all guideline statements, 715 (60.9%) were followed by at least one reference. Other characteristics about word frequencies in these statements are depicted in online supplemental file S2.

**Table 1:** Characteristics and details of diabetes guidelines included in analysis

Title of guideline	Year	Publishing association	Topic	Eligible statements	Eligible rec.’s	Sampled statements	Related rec.’s*
ADA Standards of Care in Diabetes—2024	2024	American Diabetes Association	General diabetes care	898	94	230	70
Section 3: Prevention or Delay of Diabetes and Associated Comorbidities^21^			Prevention of DM and treatment of prediabetes	93	12	17	6
Section 5: Facilitating Positive Health Behaviours and Well-being to Improve Health Outcomes^22^			Lifestyle management for diabetes	558	49	147	42
Section 8: Obesity and Weight Management for the Prevention and Treatment of Type 2 Diabetes^23^			Treatment of obesity	59	13	16	10
Section 10: Cardiovascular Disease and Risk Management^24^			Cardiovascular risk and diabetes	108	10	29	7
Section 13: Older Adults^25^			Diabetes in geriatric population	80	10	21	5
2023 ESC Guidelines for the management of cardiovascular disease in patients with diabetes^26^	2023	European Society of Cardiology	Cardiovascular risk and diabetes	131	19	30	13
Global guideline for Type 2 Diabetes^28^	2014	International Diabetes Federation	General diabetes care	30	13	9	N/A
Managing older people with type 2 diabetes. Global guideline.^27^	2013	International Diabetes Federation	Diabetes in geriatric population	116	55	31	N/A

* These are action recommendations supported by the sampled guideline statements. There are N/A’s in this column because some guidelines were not structured in a way that we could directly associate guideline statements with single action recommendation items. The 2023 ESC guideline is an updated version of 2019 ESC Guidelines on Diabetes, Pre-Diabetes and Cardiovascular Diseases^32^ and the 2019 version was co-developed by ESC and EASD.

ADA, American Diabetes Association; DM, diabetes mellitus; EASD, European Association for the Study of Diabetes; ESC, European Society of Cardiology; N/A, not applicable; rec., action recommendation.

Among the 300 sampled statements, 114 (38.0%) were causal, of which 15 (13.2%) were weak, 23 (20.2%) were moderate, and 76 (66.7%) were strong causal statements. Table 2 provides details about all the ratings.

**Table 2:** Strength of causation ratings for guideline statements

Level of causation	Rating	No.	% in category	% in total
Causal		114	100%	38.0%
Weak	1	15	13.2%	5.00%
Moderate	2	23	20.2%	7.67%
Strong	3	76	66.7%	25.3%
Non-causal		170	100%	56.7%
Correlational	0	37	21.8%	12.3%
No relationship	-1	133	78.2%	44.3%
Ineligible	N/A	16	N/A	5.33%
Total		300		100%

% in category takes the total number of sentences in this category (causal or non-causal) as denominator.

N/A, not applicable.

Following statement sampling, we extracted 83 guideline recommendations that were supported by these guideline statements. Of them, 27 (32.5%) items had one guideline statement followed, 18 (21.7%) had two statements and 14 (16.9%) had three statements (online supplemental file S2, table S2.3). About one-third (29, 34.9%) of the recommendations could not be effectively supported by any of the surrounding statements in terms of expressed strength of causation (table 3). Examples of this type of causal language jump are provided in table 4.

**Table 3:** Causation-dependence of action recommendations and alignment

Causation dependence	Rating	No.	No. eff. supported	No. ineff. supported
Guideline rec.		83 (100%)	54 (65.1%)	29 (34.9%)
Strong	3	24 (28.9%)	18 (21.7%)	6 (7.23%)
Moderate	2	23 (27.7%)	13 (15.7%)	10 (12.0%)
Weak	1	19 (22.9%)	6 (7.23%)	13 (15.7%)
None	0	17 (20.5%)	17 (20.5%)	0 (0.00%)
OS Abstract rec.		38 (100%)	35 (92.1%)	3 (7.89%)
Strong	3	8 (21.1%)	7 (18.4%)	1 (2.63%)
Moderate	2	16 (42.1%)	14 (36.8%)	2 (5.26%)
Weak	1	4 (10.5%)	4 (10.5%)	0 (0.00%)
None	0	10 (26.3%)	10 (26.3%)	0 (0.00%)
OS Main text rec.		72 (100%)	65 (90.3%)	7 (9.72%)
Strong	3	17 (23.6%)	14 (19.4%)	3 (4.17%)
Moderate	2	32 (44.4%)	29 (40.3%)	3 (4.17%)
Weak	1	18 (25.0%)	17 (23.6%)	1 (1.39%)
None	0	5 (6.94%)	5 (6.94%)	0 (0.00%)

Total number of recommendations excludes some studies where no recommendations nor conclusive sentences existed in abstracts or main texts. Percentages take the total number of recommendations in each corpus as denominator.

eff., effectively; ineff., ineffectively; OS, original study; rec., recommendation.

**Table 4:** Different types of causal language jump and non-alignment within and beyond guidelines

No.	Text (jump to)	Rating	Text (jump from)	Rating
	Jump type 1a*.* Causal language jump between guideline recommendations and guideline statements			
1a-1	*Guideline recommendation:* Principles of motivational interviewing should be considered to induce behavioural changes.(ESC2023 p. 4082; rec. table 16 item 4)	Moderate dependence	*Guideline statement with highest causation strength rating*: Perceived susceptibility to illness and the anticipated severity of the consequences are also prominent components of patients’ motivation. (ESC2023 p. 4082; par. 6 sent. 3)	No relationship
1a-2	*Guideline recommendation:* Provide an increased level of support for people with diabetes and serious mental illness through enhanced monitoring of and assistance with diabetes self-management behaviours.(ADA2024 Chr5 p. S96; rec. 5.46)	Moderate dependence	*Guideline statement with highest causation strength rating*: Disordered thinking and judgement can be expected to make it difficult to engage in behaviour that reduces risk factors for type 2 diabetes, such as restrained eating for weight management.(ADA2024 Chr5 p. S96; col. 2 sent. 3)	Weak causation
1a-3	*Guideline recommendation:* Pre-diabetes is associated with heightened cardiovascular risk; therefore, screening for and treatment of modifiable risk factors for cardiovascular disease are suggested.(ADA2024 Chr3 p. S47; rec. 3.9)	Strong dependence	*Guideline statement with highest causation strength rating:* Of note, the years immediately following smoking cessation may represent a time of increased risk for diabetes (103–105), and individuals should be monitored for diabetes development and receive evidence-based lifestyle behaviour change for diabetes prevention described in this section. (ADA2024 Chr3 p.S47; col. 1 par.2 sent. 3)	Correlational but not causal
	Jump type 1b. Causal language jump between guideline recommendations and guideline statements: when multiple statements support one recommendation			
1b-1	*Guideline recommendation:* Diabetes care teams should implement psychosocial screening protocols for general and diabetes-related mood concerns as well as other topics such as stress, quality of life, available resources (financial, social, family and emotional), and/or psychiatric history. Screening should occur at least annually or when there is a change in disease, treatment or life circumstances. (ADA2024 Chr5 p. S91 rec. 5.36)	Moderate dependence	*All rated guideline statements related to this item:* Topics to screen for may include, but are not limited to, attitudes about diabetes, expectations for treatment and outcomes (especially related to starting a new treatment or technology), general and diabetes-related mood, stress, and/or quality of life (eg, diabetes distress, depressive symptoms, anxiety symptoms, and/or fear of hypoglycaemia), available resources (financial, social, family and emotional), and/or psychiatric history. (ADA2024 Chr5 p. S92; col. 2 par. 2 sent. 2)	No relationship
			Thus, screening for SDOH (eg, loss of employment, birth of a child, or other family-based stresses) should also be incorporated into routine care (423).(ADA2024 Chr5 p. S92; col. 3 par. 1 sent. 2)	No relationship
	Jump type 2a. Causal language jump between guideline statements and original studies (not accounting for content alignment)			
2a-1	*Guideline statement:* It is known that smokeless tobacco products, such as dip and chew, pose an increased risk for CVD (348).(ADA2024 Chr5 p. S91; col. 1 sent. 2)	Strong causation	*Original study sentence with highest rating*: Promoting ST product use as a way for smokers to reduce risk for smoking-related diseases is not appropriate. (Piano 2010^33^ p. 1539; col. 2 par. 4 sent. 6)	Weak causation, partially aligned in contents
2a-2	*Guideline statement:* Physical consequences of pain include:…; Depression, sleep deprivation and worry(303,304).(IDF-O p. 75; col. 2 par. 3 sent. 2)	Strong causation	*Original study sentence with highest rating*: Pain can lead to sleep deprivation, which can decrease pain thresholds, limit the amount of daytime energy and increase the incidence and severity of depression and mood disturbances. (Hanks-Bell 2004^34^ p. 3; par. 1 sent. 4)	Moderate causation, fully aligned in contents
2a-3	*Guideline statement:* A cluster randomised trial found statistically significant increases in quit rates and long-term abstinence rates (6 months) when smoking cessation interventions were offered through diabetes education clinics, regardless of motivation to quit at baseline (360).(ADA2024 Chr5 p. S91; col. 1 par. 3 sent. 2)	Strong causation	*Original study sentence with highest rating:* Implementation of OMSC in diabetes education programmes was associated with a near quadrupling of the likelihood that smokers with type 2 diabetes or pre-diabetes would achieve long-term abstinence. (Reid 2018^35^ p. 410; col. 3 par. 2 sent. 2)	Moderate causation, fully aligned in contents
	Jump type 2b. Causal language jump between guideline statements and original studies (accounting for content alignment)			
2b-1	*Guideline statement:* Malnutrition is associated with longer length of stay in hospital and increased mortality(48), is a strong predictor of readmission and is associated with pressure ulcers, delirium and depression(49).(IDF-O p. 20; col. 1 sent. 3)	Moderate causation	*Original study sentence with highest rating:* In conclusion, overweight status in an 80-year-old population was found to be associated with longevity and underweight status with short life. (Takata 2007^36^ p. 916; col. 2 par. 3 sent. 1)	Weak causation, partially aligned in contents
2b-2	*Guideline statement:* While post-cessation weight gain is an identified issue, studies have found that an average weight gain of 3–5 kg does not necessarily persist long term or diminish the substantial cardiovascular benefit realised from smoking cessation (337).(ADA2024 Chr5 p. S90; col. 3 par. 2 sent. 2)	Strong causation	*Original study sentence with highest rating:* both active and passive smoking are associated with increased risk of incident type 2 diabetes. (Pan 2015^37^ p. 964; col. 1 par. 2 sent. 1)	Moderate causation, not aligned at all in contents
2b-3	*Guideline statement:* People with CVD and T2DM are encouraged to reduce sodium intake, as this may reduce systolic BP by, on average, 5.8 mm Hg in hypertensive patients and 1.9 mm Hg in normotensive patients. (ESC2023 p. 4062; col. 2 par. 3 sent. 1)	Moderate causation	*Original study sentence with highest rating:* decreasing sodium intake reduces blood pressure in those with diabetes. (Evert 2013^38^ p. S133; col. 1 par. 3 sent. 2)	Strong causation, partially aligned in contents
	Jump type 2c: Causal language jump between guideline statements and original studies: when a statement is supported by multiple studies			
2c-1	*Guideline statement:* The ADDITION (Anglo-Danish-Dutch Study of Intensive Treatment in People with Screen Detected Diabetes in Primary Care) trial showed that microvascular or macrovascular events were not significantly reduced after 5 or 10 years (17% and 13% reduction, respectively), while intervention only slightly improved HbA1c (364,365).(ESC2023 p. 4082; col. 2 par. 2 sent. 2)	Strong causation	*All cited original study sentences*: When compared with routine care, an intervention to promote target-driven, intensive management of patients with type 2 diabetes detected by screening was associated with small increases in the prescription of drugs and improvements in cardiovascular risk factors, but was not associated with significant reductions in the incidence of cardiovascular events or death over 5 years (Griffin 2011^39^ p. 165; col. 2 par. 6 sent. 1)	Moderate causation, fully align in content
			The non-significant 17% lower risk of cardiovascular events from the 5-year timepoint was attenuated to 13% at 10 years, and this finding remained non-significant (Griffin 2019^40^ p. 932; col. 2 par. 3 sent. 2)	No relationship, alignment not applicable

ADA2024, American Diabetes Association 2024 guideline; BP, blood pressure; Chr, chapter; col., column; CVD, cardiovascular disease; ESC2023, 2023 European Society of Cardiology guideline; HbA1c, glycated hemoglobin A1c; IDF-O, International Diabetes Federation Guideline for Older Adults; OMSC, the Ottawa Model for Smoking Cessation; p., page; par., paragraph; rec., recommendation; SDOH, social determinants of health; sent., sentence; ST, smokeless tobacco; T2DM, type 2 diabetes mellitus.

### Original study characteristics

There were 191 original studies cited to support causal statements. Two studies were excluded from the analysis because we could not retrieve the full text. Study information and characteristics can be found in online supplemental file S3. Of these studies, most were from the USA (109, 57.1%), published in Diabetes Care (29, 15.2%) or NEJM (14, 7.33%), and published between 2017 and 2022 (93, 48.7%). The articles received a median citation number of 232.0 (IQR 51.0–659.0), and 22.20 citations per year (IQR 8.75–68.10). Original studies were mainly randomised controlled trials (RCT, 52, 27.2%), followed by systematic reviews (with meta-analysis, 40, 20.9%) that mainly consisted of RCTs as well. 20 (10.5%) studies were secondary analyses or follow-up studies of an RCT, and only 15 (7.85%) were observational. All the 191 studies were included in the assessment of original study main texts; after sampling, 97 studies corresponding to 97 guideline statements were further analysed for abstracts, action recommendations and TTE components.

### Alignment between original studies and guidelines

Of the 114 causal guideline statements, 30 (26.3%) were not directly followed by any reference, and 12 (10.5%) failed to be supported by any original studies even in the surrounding statements. Among the rest of them, 46 (40.4%) were supported by one original study, 18 (15.8%) were supported by two, 7 (6.14%) were supported by three and 13 (11.4%) were supported by four or more studies (online supplemental file S2, table S2.2). In sum, these causal statements had a median of 1 original study (IQR 0–2, min. 0, max. 13, mean 1.64).

**Figure 2:**
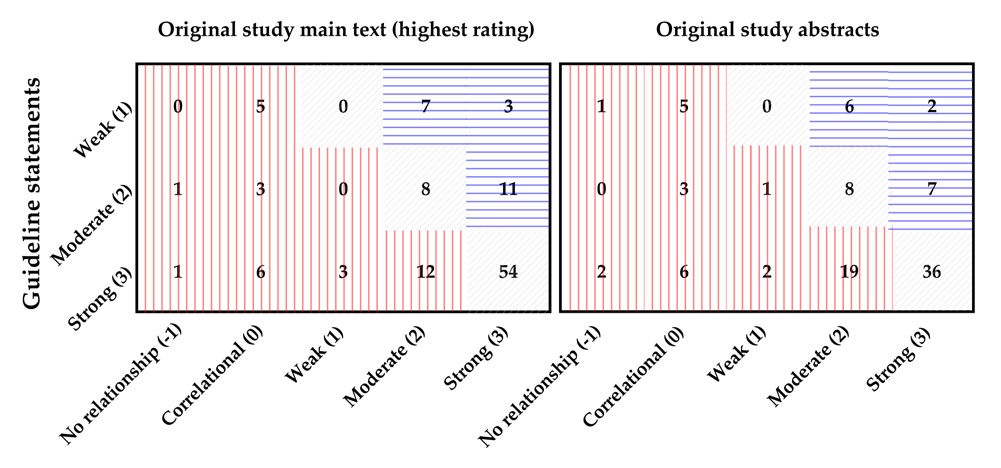
Alignment of causation strength ratings in both abstracts and main texts. Left panel: Alignment of causation ratings between causal guideline statements and main-text conclusive sentences with the highest ratings from their associated original studies, N=114; Right panel: Alignment of causation ratings between causal guideline statements and abstract conclusive sentences from their associated original studies, N=98 because some original studies do not have an eligible abstract; Numbers in cells refer to the number of items (sentences), numbers in axis labels refer to causation ratings. Red cells indicate non-alignment/causal language jumps. Cells filled with red vertical lines indicate items with lower ratings in original studies but higher ones in guideline statements; blue horizontal lines indicate items with higher ratings in original studies and lower ones in guideline statements; grey diagonal lines indicate items with the same rating in both guideline statements and original studies.

We checked whether causal language jumps were present by comparing the causation ratings in the original studies and in the guideline statements they support (figures2  3). Taking content alignment into consideration, 47.4% (54/114) of the guideline statements could not be effectively supported by any of the associated references and exhibited jumps; even without accounting for content alignment but only comparing the strength of causation, there were still 27.2% (31/114) of the statements with a causation rating higher than any of their references. This implied potential non-alignment and causal language jumps when passing evidence from the original studies to the guideline statements. In table 4, we also illustrate different situations where a causal language jump was identified.

**Figure 3:**
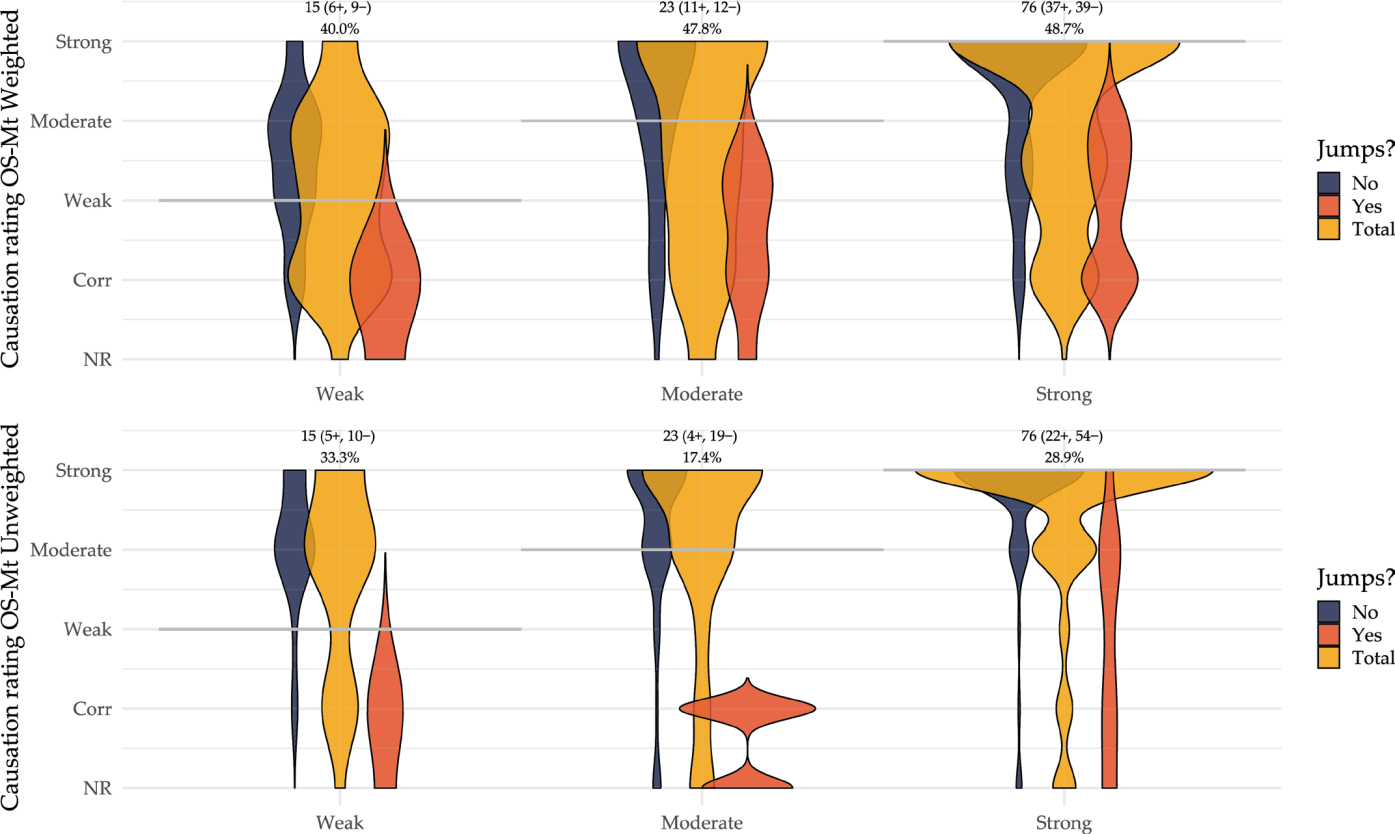
Alignment of causation strength ratings. Top panel: Alignment of causation strength between guideline statements and original study main text conclusive sentences, weighted by content alignment ratings; Bottom panel: Alignment of causation strength between guideline statements and original study main text conclusive sentences, unweighted, original ratings. Grey horizontal lines indicate the assumed lowest causation strength required from original studies for each category of guideline statement causation ratings. Numbers and percentages above the panels indicate the total count of guideline statements in each category, and the percentage of causal language jump. NR, no relationship; Corr, correlational; OS-Mt, original study main text conclusive sentence; ‘−’, causal language jump not present; ‘+’, causal language jump present.

The action recommendations contained in the original study abstracts and main texts were also similarly evaluated (table 3). Half of the abstracts of original studies and two-thirds of the main texts made action recommendations, while more than 90% of the recommendations in both abstracts and main texts were effectively supported by the conclusions of that original study or did not need evidence support. This differed from the situation in guideline recommendations, implying that guideline recommendations would have required more evidence to support.

### Reporting quality of TTE components

**Figure 4:**
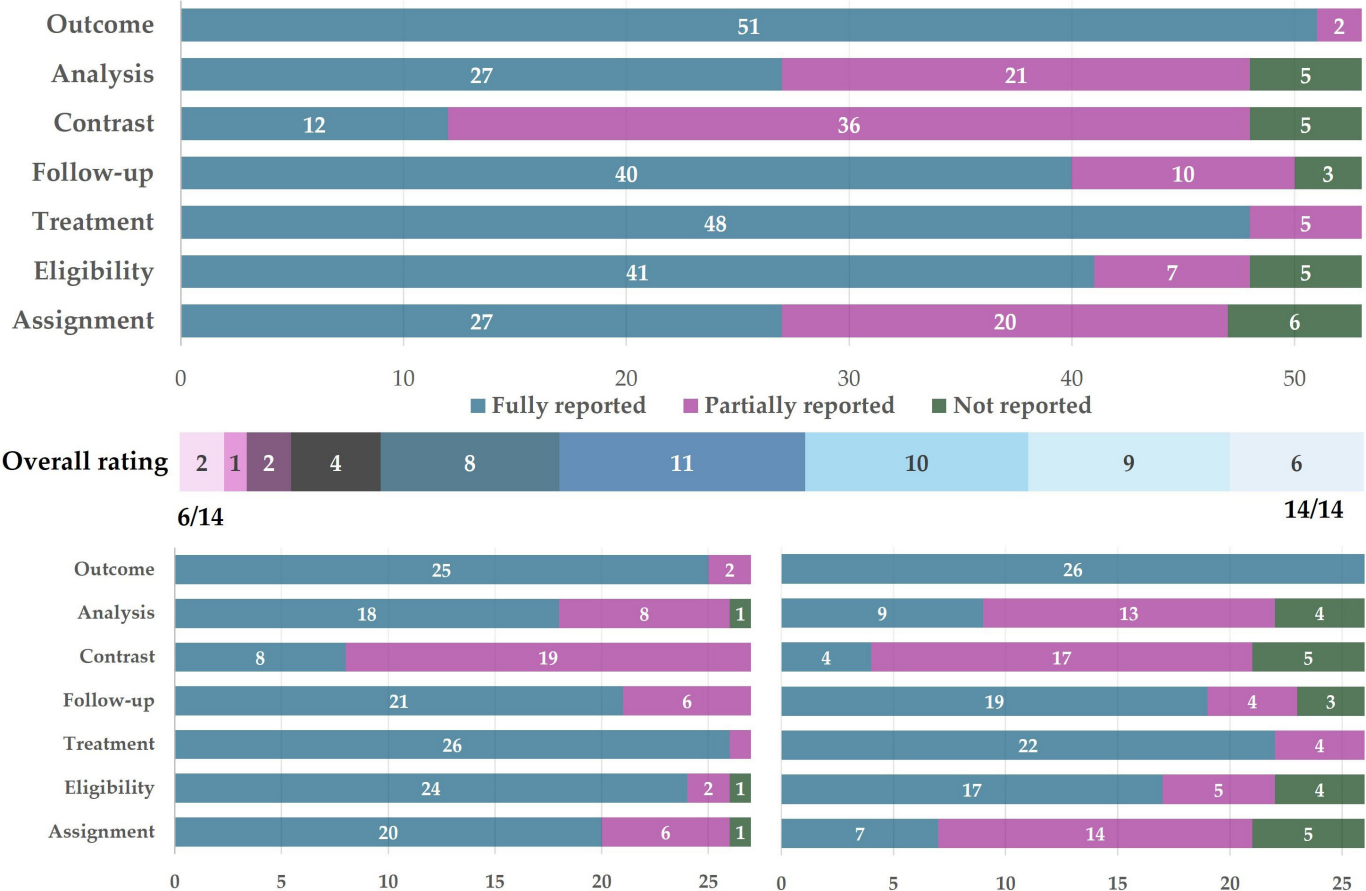
Reporting quality of TTE components. The upper panel illustrates the reporting quality of seven TTE components, and the middle panel gives the overall quality, calculated as the sum of all seven TTE component ratings each ranged from 0 (not reported) to 2 (fully reported). The lower panel gives the details of TTE ratings stratified by study types (lower-left panel for RCT studies, lower-right for non-RCT studies). RCT, randomised controlled trial; TTE, target trial emulation.

From all the 97 extracted and further analysed studies, 53 were primary studies (trials or observational studies) and were eligible for TTE component reporting quality rating. Figure 4 illustrates the distribution of ratings. Of the seven TTE components, treatment strategy and outcome assessment had the highest reporting quality, whereas causal contrast, analysis plan and assignment procedure had lower reporting quality. Most studies did not clearly state their causal estimands in the study aims, and implementation details for randomisation or covariate adjustment/matching were often not fully given.

The reporting quality differed by study type (figure 4, lower panel). There was a clear gap between the overall reporting quality scores in RCT (median 12.0, IQR 11.0–13.0) and non-RCT studies (median 10.0, IQR 9.00–11.0; Wilcoxon test: location shift 2.00 (95% CI 1.00 to 3.00), *W*=556.5, p=0.0002). Nevertheless, even in clinical trials, the TTE components were not reported at perfect quality.

### Causal linking words

We finally characterised the pattern of causal linking word use in both guidelines and original studies. The use of causal linking words to express causation varies between guidelines and original studies. In online supplemental file S4, we displayed the most used causal linking words in guideline statements and original studies, in which ‘benefit’ was most used in guidelines but ‘associated’ took the first place in original studies. ‘Associated’ ranked only fourth by frequencies of all causal linking words in guideline statements.

## Discussion

### Interpretation of main findings

In this study, we investigated the use of causal language in selected diabetes guidelines and assessed whether the strength of causation or the dependence to causation was stronger than that in the references they relied on, a phenomenon we called ‘causal language jump’. We revealed that causal language jumps and the non-alignment of expressed strength of causation were common (27.2% of the statements, or 47.4% when accounting for content alignment) between original studies and guideline statements that cited them, and also common (34.9% of the recommendations) between guideline statements and action recommendations that should be supported by the statements.

The frequent presence of causal language jumps in guidelines does not necessarily imply that guideline makers made mistakes or that someone should be accused of. For example, despite weaker causation expressed in the cited original studies, a stronger causal conclusion may be warranted when interpreting multiple studies together or in conjunction with other evidence. These jumps must still be made, however, explicitly and on the basis of good causal inference, in the same way as that a single study must make explicit arguments to justify and warrant causal conclusions. Causal language jumps are problematic when they are not warranted (overinterpreting results) or rely on non-transparent reasoning. Though, in this manuscript, we cannot differentiate between ‘safe’ and ‘dangerous’ jumps, the prevalence of either type of jump is worth concerning. This also implies that there is not, and should not be, a universal criterion of the threshold about the frequency and presence of causal language jumps. The underlying reasons and the consequences of these jumps could differ, and such reasons and consequences should be judged and discussed with domain experts, often guideline makers, extensively and thoroughly. This is also true for the improvement of ‘dangerous jumps’ and more transparent reporting of ‘safe but unspoken jumps’. Below, we discussed some possible reasons for a jump we observed from the diabetes guidelines examined in this study and gave a picture of how these jumps were different and how we should think through them.

### Safe and dangerous jumps of different reasons

Causal language jumps may be present because the strength of causation is hard to determine or could easily be misinterpreted. Controversies about the semantics of different causal/associational words largely exist^10^: there may even be multiple valid interpretations of one causal sentence. Even for the same causal estimate, two researchers may still choose different causal linking words that agree with their perceptions the most to phrase a statement. While experienced guideline makers may already notice this, the semantic vagueness of causal expressions may still hinder them from getting accurate information. In our study, despite having clear criteria (online supplemental file S2) and raters specialised in causal inference, divergence between two or more raters was still common. In particular, the strength of causation was harder to judge compared with the presence or absence of causation, as a reflection of subtleties rather than huge differences in the interpretation of the same sentence.

Another consideration is that original studies with causal intentions or aims often avoid using causal words when concluding. It is common practice in epidemiology to prefer associational language over causal language,^3^ putting the onus on guideline makers to decide whether a causal language jump is warranted. The word ‘associated’ particularly exhibits this problem, as it was even ranked the first across all possible causal linking words in original study sentences (online supplemental file S4), same as that in a previous study.^7^ That researchers conclude their results as an ‘association’ even in a trial means that they are less likely to analyse potential biases and quantify postrandomisation issues.^4^ However, guideline makers still tended to consider the evidence carried by these studies as stronger causal or as more confident ones, and some jumps were thus made. Moreover, original studies do not necessarily need to have clear implications for clinical practice, while guidelines are required to do so. Therefore, it may be more necessary for guideline recommendations to make causal language jumps or integrate other domain-specific knowledge to provide definitive conclusions that can be directly used in practice.

The causation dependence of about one-third of the action recommendations was stronger than (any of) the corresponding supporting statements. This result was similar to that found in Haber *et al*,^7^ although their evaluation considered ‘jumps’ for single studies with observational design and was not directly comparable to our results. Action recommendations in guidelines were often made not only based on original studies cited in the current single guideline, but also on previous experience, expertise and other subject-matter knowledge gained from practice and education. These deviations from the original studies may affect the causation-dependence of recommendations and further induce causal language jumps we observed. Incorporating this causal knowledge outside the range of cited original studies is unavoidable and vital and should be allowed; however, it would be better that scholars with expertise in causal inference and familiar with the disease-specific knowledge participate in this process and help on a safer ‘landing’ after this jump.

There are also cases where some studies were indeed incorrectly interpreted or read, and the guideline statements based on these studies thus used incorrect information, including causal information. This happened not only for expressed and perceived causation, but also for the alignment of contents, for example, a specific weight loss goal of 5%–7% was given in guidelines^22^ while the original studies stated weight loss was effective to reduce diabetes incidence but did not give an optimal goal of weight loss.^31^ The proportion of causal language jumps doubled after accounting for content alignment compared with the raw causation ratings, implying this could happen more often than supposed.

### Other considerations and suggestions

It is possible that guideline makers intended to cite multiple evidence pieces each of weaker causal strengths to support a stronger causal claim, because these pieces could express related information and synthesise into stronger evidence. We named such a situation ‘joint support’. About one-fourth of jumps in guideline statements and half of jumps in action recommendations could be subject to such possibility because they were supported by multiple evidence pieces (online supplemental file S2; table S2-1, S2-2). Some of the jumps reported could thus actually be a ‘joint support’ that was justified and should have been classified as ‘non-jumps’ or ‘very safe jumps’. This justification, however, happens silently and is based on decisions and assessments of background knowledge not directly accessible to the readers. It would be better for guideline developers to realise, if they refuse to label joint support situations as jumps, that such justifications and reasoning should still be reported transparently and made explicitly, as all other observed causal language jumps should be.

Lastly, the suboptimal reporting quality of TTE components adds to the ambiguity of causal information. Evaluation of the quality of the original studies will be harder if some of the TTE components were not reported or insufficiently reported, and this could also potentially lead to causal language jumps on the ‘causation flows’ when guideline makers do not read the evidence as intended. Not all the primary studies included in our study reported their TTE components in detail. Most, for example, did not explicitly state their estimands or causal contrasts. This was unexpected given the importance of having a clearly expressed causal question and/or estimand and would only make the causal claims in the original studies or in guidelines harder to be evaluated. The distribution of TTE component ratings in this study was basically comparable to that reported in Smit *et al*,^30^ with the exceptions that the eligibility criteria were reported at a worse level, and the treatment strategies were better reported. These shifts may come from the clinical trials in this study, while Smit *et al* analysed only observational studies dedicated to causal inference.

Going one step further beyond only guidelines and causal ‘language’ jump, we assert that each time a causal conclusion is made based on non-causal evidence, a certain ‘jump’ has to be made. Instead of a causal language jump, we may call this a ‘causal jump’ without the word ‘language’. In a full causation flow starting at observed data which by nature contains no causation, a causal jump will thus always be required somewhere on the path if the endpoint consists of causal statements. To make this jump valid, one must provide justified causal reasoning and, if necessary, plausible causal assumption sets. Claiming a causal conclusion based on observed data from either an RCT or a cohort also exhibits a jump and requires such reasoning and assumptions.

Back to guidelines that almost always claim causation, there are several options when making such claims: either do not make jumps and reference original studies which express causation precisely, or make only safe jumps while providing their reasoning why non-causal research they cite and non-causal sentences they write is able to support causal conclusions. To achieve this, we suggest that researchers with causal inference expertise be always invited as part of a guideline development working group, who helps justify or suppress the jumps and differentiates between safe and dangerous ones. Transparent reporting of any decisions and reasoning when making a jump is necessary if not avoidable, for example, when a recommendation must be actionable and provide detailed suggestions from domain expertise. In the end, we should use the synergy between different expertise and push guidelines towards an ideal status: safe jumps should be reported and justified as transparently as possible, and dangerous jumps should be eliminated.

### Limitations

This study had some limitations. First, we only chose to assess non-pharmacological statements and recommendations in (type 2) diabetes management. Due to our domain-specific knowledge sets and related working experience, we chose to restrict our study to this setting. Second, not all the original studies were fully assessed for TTE reporting due to their large number. Although the assessed studies were chosen randomly, there could indeed be studies of better reporting quality in unsampled references. Third, we assumed that different original studies used to support one guideline statement were independent, and therefore used the study with the strongest causal claim to assess causal jumps. It is possible that several studies, each with weaker but related evidence, could be synthesised into stronger evidence that suffices to support a stronger causal statement. We also assumed such independence between several guideline statements that are used to support an action recommendation item. However, as explained in the discussion, it is not yet possible to look into this ‘joint support’ problem at only the semantic level. The assessment of contents and domain-specific knowledge is beyond the scope of this article, and beyond the capacity of average users and readers of a guideline. These ‘joint support’ should thus be reported and justified by guideline makers explicitly, as all other observed causal language jumps should be.

## Conclusions

Our study revealed that non-alignment between strength of causation and causal language jumps in the causation flows were common in clinical practice guidelines about diabetes management. One-third of the guideline statements were causal, and more than one-fourth of them made causal language jumps in terms of causation strength, compared with the original studies. The observed jumps can partially be attributed to suboptimal status of causal and associational language use in clinical studies and epidemiology. While causal language jumps are sometimes inevitable to make, good and responsible practice of causal inference can be helpful for a valid and justified jump.

## Supplementary material

10.1136/bmjopen-2025-109205 online supplemental file 1

## Data Availability

Data are available on reasonable request. All data relevant to the study are included in the article or uploaded as supplementary information.
