# Supplementary material for: Causal language jumps and non-alignments between clinical practice guidelines and original studies: a systematic evaluation of diabetes guidelines and their cited evidence
Source: BMJ Open. 2026 Feb 5;16(2):e109205. doi: 10.1136/bmjopen-2025-109205 (PMC12878190; doi:10.1136/bmjopen-2025-109205)
Supplement: online supplemental file 1 [file bmjopen-16-2-s001.pdf]

Causal language jumps and non-alignments between clinical practice guidelines and original studies: a systematic evaluation of diabetes guidelines and their cited evidence

Supplementary data

27 November 2025

File contents

File S1 Details of scales used for rating

File S2 Guideline and guideline statement characteristics

File S3 Original study characteristics

File S4 Causal linking words in guidelines and original studies

Contents

File S1 Details of scales used for rating **2**
S1.1 Scale for causation strength 2
S1.2 Scale for recommendation causation-dependence 4
S1.3 Scale for target trial emulation components 5
S1.4 Examples for rating 10

File S2 Guideline and guideline statement characteristics **12**
S2.1 Rationale of the inclusion of selected guidelines and their sections 12
S2.2 Word use characteristics in guideline statements and recommendations . 15
S2.3 Action recommendation items and causal language jump frequencies by number of guideline statements 17
S2.4 Guideline statement and causal language jump frequencies by number of references 18
S2.5 Original study sentence characteristics 19

File S3 Original study characteristics **20**

File S4 Causal linking words in guidelines and original studies **52**

References cited in supplementary files **55**

File S1

Details of scales used for rating

This appendix contains details, rubrics and rater instructions for (1) *The Causal Implication Strength Rating Scale* and (2) the Target Trial Emulation (TTE) component reporting quality scale. This was used in the rating of guideline statements, original study sentences, and reporting quality.

S1.1 Scale for causation strength

Table S1.1: The Causal Implication Strength Rating Scale for causation strength.

Rating	Description	Linking word	Modifier
3<br>Strong	The linking sentence clearly implies that causality had been identified.	A linking word expressing strong explicit causation, *e.g.* "effect", "beneficial".	No weakening modifiers.

Table S1.1 (Continued)

Rating	Description	Linking word	Modifier
2 Moderate	The linking sentence mostly implies that a causal relationship was identified, but it is unclear or not possible to come to that conclusion in the absence of any causal inference.	A linking word expressing strong explicit causation, *e.g.* "effect", "beneficial".	One weakening modifier that weakens the tone or the expression of a sentence, *e.g.* "may", "could", "probably"
2 Moderate	Same as above	A linking word expressing weak or implicit causation, *e.g.* "associated", "risky".	No weakening modifiers.
1 Weak	The linking sentence might imply that a causal relationship was identified, but it is unclear or not possible to come to that conclusion in the absence of any causal inference.	A linking word expressing strong explicit causation, *e.g.* "effect", "beneficial".	Several weakening modifiers that weaken the tone or the expression of a sentence, *e.g.* "may", "could", "probably"; Or the overall tone of the sentence expresses very weak confidence about the causation implied.
1 Weak	Same as above	A linking word expressing weak or implicit causation, *e.g.* "associated", "risky".	One or more weakening modifiers that weakens the tone or the expression of a sentence, *e.g.* "may", "could", "probably"

Table S1.1 (Continued)

Rating	Description	Linking word	Modifier
0<br>None	The linking sentence does not imply in any way that a causal relationship was identified.	Note: The sentence still contains relationships between a set of variables or talks about correlation.	–
-1<br>N/A	–	Note: The sentence does not contain any variable relationships and is unrelated to correlation.	–

The description of this scale is modified from Haber et al. [1].

S1.2 Scale for recommendation causation-dependence

Table S1.2: The Causal Implication Strength Rating Scale for causation-dependence of action recommendations

Rating	Description	Explanation
3<br>Strong	The action recommendation could only be made appropriately had a causal relationship been identified.	We must make this recommendation based on new causal evidence; common knowledge sets are never sufficient to support it anymore.

Table S1.2 (Continued)

Rating	Description	Explanation
2<br>Moderate	The action recommendation most likely could only be made appropriately had a causal relationship been identified, but it is unclear or not possible to come to that recommendation in the absence of any causal inference.	We need to make this recommendation based on new evidence, along with our common knowledge sets and subject-matter knowledge.
1<br>Weak	The action recommendation may be made appropriately had a causal relationship been identified, but it is unclear or not possible to come to that recommendation in the absence of any causal inference.	We can make this recommendation with a little evidence and based on our common knowledge sets and subject-matter knowledge.
0<br>None	The action recommendation would be made appropriately in the absence of any causal relationship.	We can make this recommendation in the absence of any evidence, only based on our common knowledge sets and subject-matter knowledge.
-1<br>N/A	No action recommendation exists.	–

The description of this scale is modified from Haber et al. [1].

S1.3 Scale for target trial emulation components

Table S1.3: The reporting quality scale for target trial emulation components

TTE component	Fully reported (2/2)	Partially reported (1/2)	Not reported (0/2)
Eligibility criteria	Inclusion and exclusion criteria of participants are clearly given, or given in a protocol, prior to the description of the subsequent randomization and treatment	Inclusion and exclusion criteria are somewhat given, not clear enough, or given in brief. It requires additional judgements if one were to define the study population with the information given.	Only inclusion or exclusion criteria are given, and are in vast brief. Or that the eligibility criteria are reported along with participant selection or participant demographics/characteristics. It is very hard to define a study population.
Treatment strategies	[Interventional study]: treatment strategy is clearly given, with its time span, run-in or initiation, and procedures, which is replicable.	[Interventional study]: treatment strategy is given, but is not very detailed; time span, planned intervention sessions, monitoring, or some procedures could be not clear enough.	[Interventional study]: treatment strategy is very roughly described and cannot be replicated if asked to do so at all, or there is barrier from understanding how a treatment is carried out.
	[Observational study]: the definition of the exposure, as well as how and when it is measured, preferably also time span, is clearly given.	[Observational study]: at least one aspect mentioned in the left side is not clear enough or not reported.	[Observational study]: the definition of the exposure and how and when it is measured are unclear.

Table S1.3 (Continued)

TTE component	Fully reported (2/2)	Partially reported (1/2)	Not reported (0/2)
Assignment procedures	[Randomized study]: The randomization procedure, including how block/stratum is constructed and with which method randomization is performed, needs to be described clearly.	[Randomized study]: The randomization is mentioned, but the specifications are not reported in detail.	[Randomized study]: The study only claims that this is a randomized study, but any further information is not covered by the article text.
	[Other study]: Covariate adjustment and between-group balance considerations before and after the [emulated] randomization time point should be discussed and appropriately conducted and reported.	Covariate adjustment and considerations about how to make groups comparable is mentioned but not in detail; rationale about the variables chosen is completely not given.	Covariate adjustment for group balance or the construction of a theoretical control for single-arm trials are not given or are given but are vast brief and very unjustified.
Follow-up period	The time zero, time span of follow-up, and the definition of the end of follow-up (if applicable) and the endpoint events that trigger ending follow-up, are clearly reported. Preferably, the time zero is explicitly stated in the study text.	The time span of follow-up is reported, but some information is missing, and it could be hard to replicate this study or reconstruct time at risks from raw data if one were to do so.	The time span, risk period, or related considerations, are not reported. It is confused how the authors measured variables and/or decided to start/end follow-up.

Table S1.3 (Continued)

TTE component	Fully reported (2/2)	Partially reported (1/2)	Not reported (0/2)
Outcome of interest	The outcomes of interest are clearly defined. This includes the operationalized definition, how to measure it, and preferably the associated time span or a specific time point at which outcomes are measured.	The outcomes of interest are partially reported; or only the primary outcome is given, while the information about the measurement of other outcomes is missing. It is hard to replicate this study's outcome measurement and calculation using information given.	The outcomes are very vaguely defined. It is nearly impossible to think about the procedure of outcome measurements and comparisons.
Causal contrast	The causal contrast, or the estimand, is clearly stated in the research question. This often appears at the very end of an introduction section of an article. The causal contrast includes: at least two treatment levels to be contrasted, an outcome that is clearly stated, time period, population, and other components.	The causal contrast or the estimand is somewhat expressed in the research question. There is not a clear sentence about the effect of interest, but traditional components – PICOS, are mostly given.	Although the study should be of a causal nature or of causal interests, the causal contrast, even the PICOS-components, are not clearly given or very badly expressed in the article.

Table S1.3 (Continued)

TTE component	Fully reported (2/2)	Partially reported (1/2)	Not reported (0/2)
Analysis plan	An analysis plan should be clearly presented, especially including 1 under which principle (ITT/PP/AT/CC) the analysis is carried out, and 2 how to deal with (emulated) post-randomization issues (loss to follow-up, nonadherence, contamination, etc.) For observational studies, adjustment for selection and losses to follow-up should be given, while ITT/PP principles need not to be mentioned.	An analysis plan is given, but at least one critical aspect is missing, such that it would be hard to replicate a complete data analysis procedure even with raw data, if only given the analysis plan reported.	The analysis plan is not given or very vague, and cannot provide any useful information that helps look into the implementation of actual data analysis. It is not possible to replicate a complete data analysis workflow as in the article.

Abbreviations: PICOS, population, intervention, control, outcome, and study type; ITT, intention-to-treat; PP, per-protocol; AT, as-treated; CC, complete-case. The 3-item scale for reporting quality was derived from Smit et al. [2].

S1.4 Examples for rating

In this section we give examples for each of the sentence ratings listed above and their rating considerations.

Example 1: guideline statement causation rating Disordered thinking and judgment can be expected to make it difficult to engage in behavior that reduces risk factors for type 2 diabetes, such as restrained eating for weight management.

- Causation rating: 1/3
- Considerations: (1) "make it difficult to engage" is a weak linking word that express some uncertainty; (2) "can be expected to" is a weakening modifier that make the sentence tone weaker with more uncertainty.

Example 2: guideline statement causation rating Potential adverse effects of antihypertensive therapy (e.g., hypotension, syncope, falls, AKI, and electrolyte abnormalities) should also be taken into account.

- Causation rating: 2/3
- Considerations: "adverse effects" is a strong causal linking word; however, "potential" is a modifier and adds to the uncertainty.

Example 3: guideline statement causation rating An RCT found that two meal-planning approaches (diabetes plate method and carbohydrate counting) were effective in helping achieve improved A1C.

- Causation rating: 3/3
- Considerations: "effective" is a strong causal linking word.

Example 4: guideline recommendation causation-dependence Monitor changes in body weight, glycemia, and lipids in adolescents and adults with diabetes who are prescribed second-generation antipsychotic medications; adjust the treatment plan accordingly, if needed.

- Causation-dependence rating: 0/3
- Considerations: This is more common sense and general practice; no novel evidence is necessary to support it.

Example 5: guideline recommendation causation-dependence Nutrition, physical activity, and behavioral therapy to achieve and maintain =5% weight loss are recommended for people with type 2 diabetes and overweight or obesity.

- Causation-dependence rating: 3/3

- Considerations: The goal of 5% weight loss is very specific and requires original studies that are dedicated to test for this percentage of weight loss to support, showing this is the optimal percentage.

Example 6: content alignment between original studies and guideline statements **[Guideline]** Therefore, other programs and modalities of behavioral counseling for diabetes prevention may also be appropriate and efficacious based on individual preferences and availability. **[Original study]** The percentage of participants retained through 18 weeks varied by age, race/ethnicity, mean weekly percentage of body weight lost, and mean weekly physical activity minutes but not by sex.

- Content alignment rating: 0/2
- Considerations: The guideline statement described the efficacy or "appropriateness", while the original study conclusive sentence provided only information on attrition for one specific treatment.

Example 7: content alignment between original studies and guideline statements **[Guideline]** This benefit was greatest in people with diabetes. **[Original study]** In high-risk patients, there are additional benefits from more intensive blood pressure lowering.

- Content alignment rating: 1/2
- Considerations: (Combining with the context) the two sentences both talked about intensive blood pressure lowering strategies; however, the second one did not talk about whether this benefit was "greatest" in patients of higher risk of diabetes.

File S2

Guideline and guideline statement characteristics

S2.1 Rationale of the inclusion of selected guidelines and their sections

In addition to the pre-defined eligibility criteria described in the Methods section, we discussed with experts in the field of diabetes and resulted in the final four sets of guidelines that were included and analyzed in our study. Here we gave detailed rationale about the inclusion of each guideline from our discussion with experts and from expert suggestions.

1. American Diabetes Association (ADA) guidelines: The ADA is the national diabetes organization in the United States and holds the world's largest diabetes meeting every year (Stated on the website). It publishes *Standards of Care* for diabetes every year, and publishes an international journal *Diabetes Care* that are cited the most in the field of diabetes according to Clarivate Journal Citation Report. Their guidelines are leading the practice of diabetes treatment and received a leading number of citations. The ADA 2024 guideline sections were the most updated version of ADA Standards of Care at the time of conducting data analysis, and were the eligible ones among all the sections.

2. European Association for the Study of Diabetes (EASD) and European Society of Cardiology (ESC) guidelines: the two associations are the European forces in the field of diabetes. They jointly published the 2007 and 2019 guideline on diabetes, and their 2023 updates. The 2019 guideline received more than 1700 citations (Web of Sciences), ranked the fifth among all diabetes-related guidelines, after the ADA guidelines of various years (Web of Sciences). The 2023 guideline is an updated version of the 2019 guideline and received more than 1000 citations

(Google Scholar).

3. International Diabetes Federation (IDF) guidelines: the IDF is a non-profit umbrella organization of 251 national diabetes associations in 158 countries and territories (IDF website) and publishes guidelines, recommendations and position statements. The included two guidelines from IDF are the most updated ones with complete references and methodology.

S2.2 Word use characteristics in guideline statements and recommendations

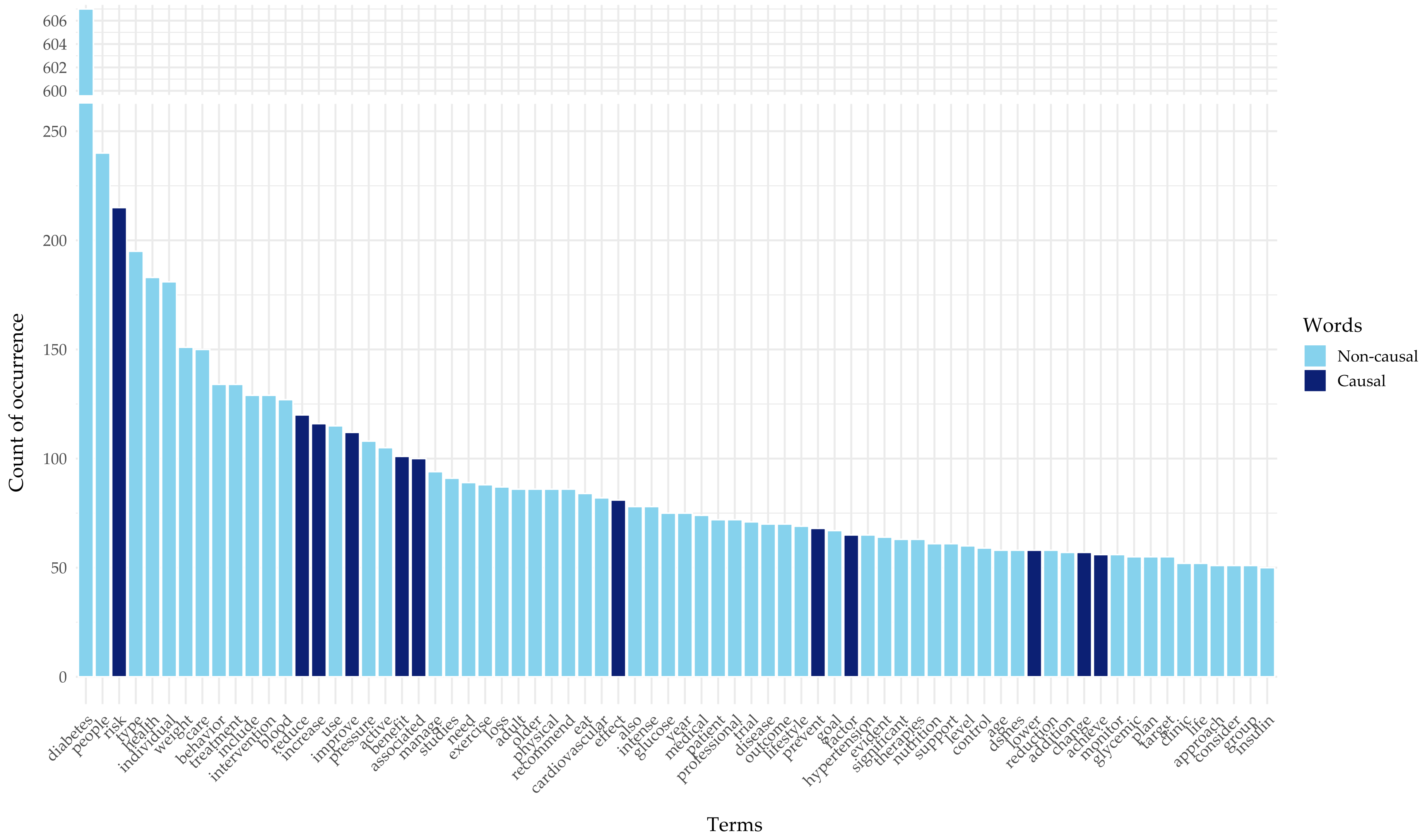

Figure S2.1: Count of occurrence of top-hit words in supporting statements. Only those appeared more than 50 times were included. Causal linking words were marked using the candidate list.

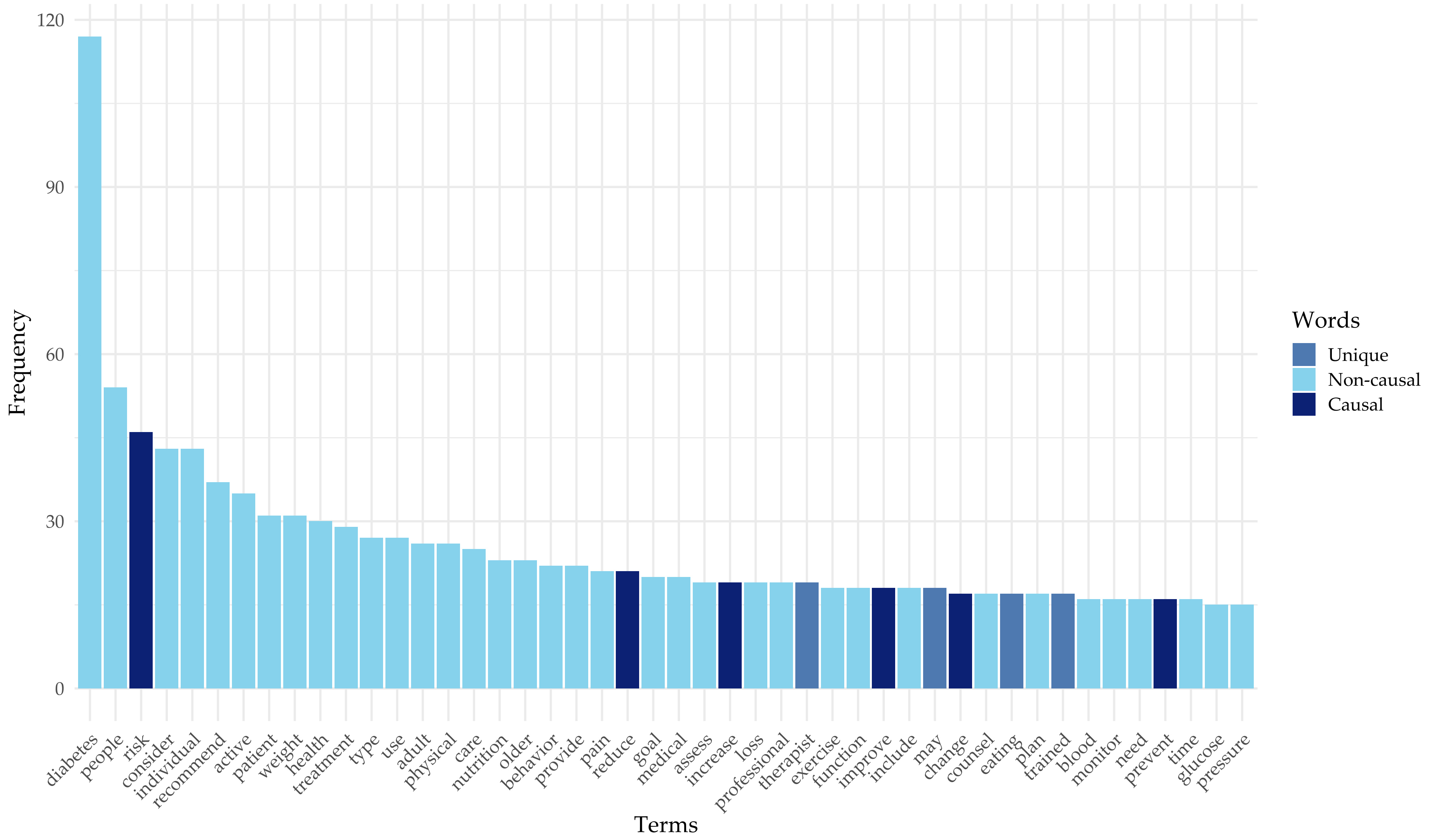

Figure S2.2: Count of occurrence of top-hit words in recommendation texts. Dark blue bars indicate candidate causal linking words; mid-blue bars for unique words in recommendation texts but not in supporting statements; and light-blue bar for other words.

S2.3 Action recommendation items and causal language jump frequencies by number of guideline statements

In Table S2.1, we provided a detailed breakdown of the frequency of action recommendation items by the number of guideline statements that follow them.

Table S2.1: Frequency of action recommendation items and jumps by number of rated guideline statements

No. of guideline statements for one rec.	1	2	3	4	5	6	7	>7
No. of rec.	27	18	14	9	5	2	4	4
Proportion in all rec. (N = 83)	32.5%	21.7%	16.9%	10.8%	6.02%	2.41%	4.82%	4.82%
No. of jumps observed according to our method	13	6	4	3	0	1	2	0
Proportion of jumps (N = 29)	44.8%	20.7%	13.8%	10.3%	0.00%	3.45%	6.90%	0.00%

rec., action recommendation item

S2.4 Guideline statement and causal language jump frequencies by number of references

In Table S2.2, we provided a detailed breakdown of the frequency of causal guideline statements by the number of references that follow them. Two thirds or our guideline statements have only one reference or no reference.

Table S2.2: Frequency of causal guideline statements and jumps by number of reference

No. of reference in statements	0	0*	1	2	3	4	>4
No. of statements	12	18	46	18	7	4	9
Proportion in causal statements (N = 114)	10.5%	15.8%	40.4%	15.8%	6.14%	3.51%	7.89%
Proportion in all statements (N = 300)	4.00%	6.00%	15.3%	6.00%	2.33%	1.33%	3.00%
No. of jumps observed, not accounting for alignment	12	3	13	3	0	0	0
Proportion of jumps observed in this category	100%	16.7%	28.3%	16.7%	0.00%	0.00%	0.00%
No. of jumps observed, accounting for alignment	12	7	20	11	2	1	1
Proportion of jumps observed in this category	100%	38.9%	43.5%	61.1%	28.6%	25.0%	11.1%

*: there is no reference following the statement itself, but a reference in its surrounding text is found to be related to this statement. See "methods: Original study selection and assessment" for details.

S2.5 Original study sentence characteristics

In Table S2.3, we illustrated the most frequently used causal linking words in different corpora, *i.e.* group of the sentences in original studies that were evaluated in our study. There are four rows since we checked one conclusive sentence and one action recommendation sentence in both abstracts and main texts of the original studies, thus forming in total four groups of sentences. For each row, we listed the top 5 frequently used causal linking words in the corresponding group to illustrate how they are different in choosing and using these words.

Table S2.3: Characteristics for sentences from original studies

Group of sentences (corpora)	Character count	Word count	Top 5 candidate causal linking words (count of occurrence)
Abstract conclusive sentences	173.0 (126.8 – 213.2)	20.0 (15.8 – 25.3)	risk (27), improve (22), significant (21), associated (20), effect (17)
Abstract action recommendations	131.0 (98.0 – 182.0)	16.0 (12.0 – 21.5)	benefit (6), improve (6), effect (6), associated (3), risk (3)
Main text conclusive sentences	160.0 (126.0 – 207.0)	19.0 (15.0 – 24.3)	associated (25), significant (22), risk (19), improve (19), effect (18)
Main text action recommendations	167.0 (129.0 – 248.0)	20.0 (14.8 – 32.3)	prevent (12), risk (11), reduce (11), effect (10), improve (9)

Note: This table summarizes the most frequently used causal linking words in the four corpora listed under “source”. The second and third column depict the median and inter-quartile range of the number of characters and words in a single sentence from the four corpora. The fourth column depicts the top 5 (candidate) causal linking words; the numbers in the parentheses are the counts of occurrence.

File S3

Original study characteristics

Table S3.1: Original study characteristics

Title	Study type*	First author	Last author	Year	Nationality	Journal	Cited by	Cited by per year	Sampled
Diabetes and Hypertension: A Position Statement by the American Diabetes Association	7	Ian H de Boer	George Bakris	2017	USA	Diabetes Care	852	106.5	Yes
Syncope, Hypotension, and Falls in the Treatment of Hypertension: Results from the Randomized Clinical Systolic Blood Pressure Intervention Trial	2	Kaycee M. Sink	Lawrence J. Fine	2018	USA	Journal of the American Geriatrics SOCIETY	96	13.7	Yes

*Code for study type: 1: randomized controlled trial (RCT), primary report; 2: RCT, secondary analysis or follow-up; 3: cohort-based observational studies; 4: other observational studies; 5: meta-analysis; 6: Previous high-level (aggregated) evidence; 7: expert-based statements; 8: others, for example single-arm self-control studies

Table S3.1 continued from previous page

Title	Study type	First author	Last author	Year	Nationality	Journal	Cited by	Cited by per year	Sampled
Blood Pressure Lowering in Type 2 Diabetes: A Systematic Review and Meta-analysis	5	Connor A. Emdin	Anushka Patel	2015	GBR	JAMA	1073	107.3	Yes
Trial of Intensive Blood-Pressure Control in Older Patients with Hypertension	1	Weili Zhang	Jun Cai	2021	CHN	NEJM	462	115.5	Yes
Effects of Intensive Blood-Pressure Control in Type 2 Diabetes Mellitus	1	The ACCORD Study Group	The ACCORD Study Group	2010	USA	NEJM	4340	289.3	Yes
Effects of intensive blood pressure lowering on cardiovascular and renal outcomes: updated systematic review and meta-analysis	5	Xinfang Xie	Anthony Rodgers	2016	CHN	The Lancet	1192	132.4	Yes
Blood Pressure Targets in Subjects With Type 2 Diabetes Mellitus/Impaired Fasting Glucose: Observations From Traditional and Bayesian Random-Effects Meta-Analyses of Randomized Trials	5	Sripal Bangalore	Franz H. Messerli	2011	USA	Circulation	592	42.3	Yes
Less-Tight versus Tight Control of Hypertension in Pregnancy	1	Laura A. Magee	Jean-Marie Moutquin	2015	CAN	NEJM	766	76.6	Yes
Effects on Blood Pressure of Reduced Dietary Sodium and the Dietary Approaches to Stop Hypertension (DASH) Diet	1	Frank M. Sacks	Jeffrey A. Cutler	2001	USA	NEJM	7179	299.1	Yes

*Code for study type: 1: randomized controlled trial (RCT), primary report; 2: RCT, secondary analysis or follow-up; 3: cohort-based observational studies; 4: other observational studies; 5: meta-analysis; 6: Previous high-level (aggregated) evidence; 7: expert-based statements; 8: others, for example single-arm self-control studies

Table S3.1 continued from previous page

Title	Study type	First author	Last author	Year	Nationality	Journal	Cited by	Cited by per year	Sampled
2014 Evidence-Based Guideline for the Management of High Blood Pressure in Adults: Report From the Panel Members Appointed to the Eighth Joint National Committee (JNC 8)	7	Paul A. James	Eduardo Ortiz	2014	USA	JAMA	11691	1062.8	Yes
Effect of structured physical activity on prevention of major mobility disability in older adults: the LIFE study randomized clinical trial.	1	Marco Pahor	Jeff D Williamson	2014	USA	JAMA	1560	141.8	Yes
Cardiovascular Effects of Intensive Lifestyle Intervention in Type 2 Diabetes	1	The Look AHEAD Research Group	The Look AHEAD Research Group	2013	USA	NEJM	3046	253.8	Yes
Association of the magnitude of weight loss and changes in physical fitness with long-term cardiovascular disease outcomes in overweight or obese people with type 2 diabetes: a post-hoc analysis of the Look AHEAD randomised clinical trial	2	The Look AHEAD Research Group	The Look AHEAD Research Group	2016	USA	The Lancet Diabetes and Endocrinology	11	1.2	Yes
Aging and physical function in type 2 diabetes: 8 years of an intensive lifestyle intervention	2	W. Jack Rejeski	Edward H. Ip	2014	USA	The Journals of Gerontology Series A	63	5.7	Yes

*Code for study type: 1: randomized controlled trial (RCT), primary report; 2: RCT, secondary analysis or follow-up; 3: cohort-based observational studies; 4: other observational studies; 5: meta-analysis; 6: Previous high-level (aggregated) evidence; 7: expert-based statements; 8: others, for example single-arm self-control studies

Table S3.1 continued from previous page

Title	Study type	First author	Last author	Year	Nationality	Journal	Cited by	Cited by per year	Sampled
Cognitive performance declines in older adults with type 1 diabetes: results from 32 years of follow-up in the DCCT and EDIC Study	2	Alan M Jacobson	John M Lachin	2021	USA	The Lancet Diabetes and Endocrinology	74	18.5	Yes
Long-term effects of lifestyle intervention or metformin on diabetes development and microvascular complications over 15-year follow-up: the Diabetes Prevention Program Outcomes Study	2	Diabetes Prevention Program Research Group	Diabetes Prevention Program Research Group	2015	USA	The Lancet Diabetes and Endocrinology	1021	102.1	Yes
Sustained reduction in the incidence of type 2 diabetes by lifestyle intervention: follow-up of the Finnish Diabetes Prevention Study	2	Jaana Lindström	Jaakko Tuomilehto	2006	FIN	The Lancet	2471	130.1	Yes
Clinical and Economic Impact of a Digital, Remotely-Delivered Intensive Behavioral Counseling Program on Medicare Beneficiaries at Risk for Diabetes and Cardiovascular Disease	3	Fang Chen	Timothy M. Dall	2016	USA	PLoS One	61	6.8	Yes
Retention Among Participants in the National Diabetes Prevention Program Lifestyle Change Program, 2012–2017	4	Michael J. Cannon	Bryce D. Smith	2020	USA	Diabetes Care	111	22.2	Yes

*Code for study type: 1: randomized controlled trial (RCT), primary report; 2: RCT, secondary analysis or follow-up; 3: cohort-based observational studies; 4: other observational studies; 5: meta-analysis; 6: Previous high-level (aggregated) evidence; 7: expert-based statements; 8: others, for example single-arm self-control studies

Table S3.1 continued from previous page

Title	Study type	First author	Last author	Year	Nationality	Journal	Cited by	Cited by per year	Sampled
Position of the Academy of Nutrition and Dietetics: the role of medical nutrition therapy and registered dietitian nutritionists in the prevention and treatment of prediabetes and type 2 diabetes.	7	Kathaleen Briggs Early	Kathleen Stanley	2018	USA	Journal of the Academy of Nutrition and Dietetics	186	26.6	Yes
The Use of Language in Diabetes Care and Education	7	Jane K. Dickinson	Martha M. Funnell	2017	USA	Diabetes Care	307	38.4	Yes
Self-management support in “real-world” settings: An empowerment-based intervention	8	Tricia S. Tang	Jacob E. Kurlander	2010	USA	Patient Education and Counseling	294	19.6	Yes
Physical Activity of Moderate Intensity and Risk of Type 2 Diabetes: A systematic review	5	Christie Y. Jeon	Rob M. van Dam	2008	USA	Diabetes Care	1149	67.6	Yes
Weight-Loss Therapy in Type 2 Diabetes: Effects of Phentermine and Topiramate Extended Release	2	W. Timothy Garvey	Barbara Troupin	2014	USA	Diabetes Care	148	13.5	Yes
Development and validity of a 2-item screen to identify families at risk for food insecurity	4	Erin R Hager	Deborah A Frank	2010	USA	Pediatrics	1239	82.6	Yes
The diabetes nutrition education study randomized controlled trial: a comparative effectiveness study of approaches to nutrition in diabetes self-management education.	1	Michael E Bowen	Russell L Rothman	2016	USA	PATIENT EDUCATION AND COUNSELING	136	15.1	Yes

*Code for study type: 1: randomized controlled trial (RCT), primary report; 2: RCT, secondary analysis or follow-up; 3: cohort-based observational studies; 4: other observational studies; 5: meta-analysis; 6: Previous high-level (aggregated) evidence; 7: expert-based statements; 8: others, for example single-arm self-control studies

Table S3.1 continued from previous page

Title	Study type	First author	Last author	Year	Nationality	Journal	Cited by	Cited by per year	Sampled
Behavioral approaches to nutrition and eating patterns for managing type 2 diabetes: A review	7	Meg G. Salvia	Paula A. Quatromoni	2023	USA	American Journal of Medicine Open	14	7	Yes
The Effect of Early Time-Restricted Eating vs Later Time-Restricted Eating on Weight Loss and Metabolic Health	5	Juanhong Liu	Feng Liu	2023	CHN	The Journal of Clinical Endocrinology & Metabolism	28	14	Yes
Efficacy of low carbohydrate diet for type 2 diabetes mellitus management: A systematic review and meta-analysis of randomized controlled trials	5	Yan Meng	Liyong Chen	2017	CHN	Diabetes Research and Clinical Practice	284	35.5	Yes
Effects of Carbohydrate Counting on Glucose Control and Quality of Life Over 24 Weeks in Adult Patients With Type 1 Diabetes on Continuous Subcutaneous Insulin Infusion: A randomized, prospective clinical trial (GIOCAR)	1	Andrea Laurenzi	Marina Scavini	2011	ITA	Diabetes Care	179	12.8	Yes
Nutrition Therapy for Adults With Diabetes or Prediabetes: A Consensus Report	6	Alison B. Evert	William S. Yancy, Jr	2019	USA	Diabetes Care	1290	215	Yes
Effects of n?3 Fatty Acid Supplements in Diabetes Mellitus	1	The ASCEND Study Collaborative Group	The ASCEND Study Collaborative Group	2018	GBR	NEJM	750	107.1	Yes

*Code for study type: 1: randomized controlled trial (RCT), primary report; 2: RCT, secondary analysis or follow-up; 3: cohort-based observational studies; 4: other observational studies; 5: meta-analysis; 6: Previous high-level (aggregated) evidence; 7: expert-based statements; 8: others, for example single-arm self-control studies

Table S3.1 continued from previous page

Title	Study type	First author	Last author	Year	Nationality	Journal	Cited by	Cited by per year	Sampled
Cardiovascular Risk Reduction with Icosapent Ethyl for Hypertriglyceridemia	1	Deepak L. Bhatt	Christie M. Ballantyne	2018	USA	NEJM	3015	430.7	Yes
A 12-week randomized clinical trial investigating the potential for sucralose to affect glucose homeostasis	1	V. Lee Grotz	J. Richard Trout	2017	USA	Regulatory Toxicology and Pharmacology	95	11.9	Yes
2018 Physical Activity Guidelines Advisory Committee Scientific Report	7	Abby C. King	Abby C. King	2018	USA	.	.	.	Yes
The Effects of Text Messages for Promoting Physical Activities in Prediabetes: A Randomized Controlled Trial	1	Prawit Bootwong	Nirun Intarut	2022	THA	Telemedicine and e-Health	9	3	Yes
Physical Activity and Mortality in Individuals With Diabetes Mellitus: A Prospective Study and Meta-analysis	5	Diewertje Sluik	Ute Nöthlings	2012	DEU	JAMA Internal Medicine	427	32.8	Yes
Physical fitness and activity changes after a 24-week soccer-based adaptation of the U.S diabetes prevention program intervention in Hispanic men	8	Jennifer K. Frediani	Felipe Lobelo	2020	USA	Progress in Cardiovascular Diseases	21	4.2	Yes
Physical Activity/Exercise and Diabetes: A Position Statement of the American Diabetes Association	7	Sheri R. Colberg	Deborah F. Tate	2016	USA	Diabetes Care	3106	345.1	Yes
Acute high-intensity interval exercise reduces the postprandial glucose response and prevalence of hyperglycaemia in patients with type 2 diabetes	8	J. B. Gillen	M. J. Gibala	2012	CAN	Diabetes, Obesity and Metabolism	398	30.6	Yes

*Code for study type: 1: randomized controlled trial (RCT), primary report; 2: RCT, secondary analysis or follow-up; 3: cohort-based observational studies; 4: other observational studies; 5: meta-analysis; 6: Previous high-level (aggregated) evidence; 7: expert-based statements; 8: others, for example single-arm self-control studies

Table S3.1 continued from previous page

Title	Study type	First author	Last author	Year	Nationality	Journal	Cited by	Cited by per year	Sampled
Clinical outcomes and glycaemic responses to different aerobic exercise training intensities in type II diabetes: a systematic review and meta-analysis	5	Aimee Grace	Neil A. Smart	2017	AUS	Cardiovascular Diabetology	195	24.4	Yes
Leisure-Time Running Reduces the Risk of Incident Type 2 Diabetes	3	Yuehan Wang	Steven N Blair	2019	NLD	The American Journal of Medicine	42	7	Yes
The health consequences of smoking—50 years of progress: a report of the Surgeon General	7	National Center for Chronic Disease Prevention and Health Promotion (US) Office on Smoking and Health	National Center for Chronic Disease Prevention and Health Promotion (US) Office on Smoking and Health	2014	USA	.	.	.	Yes
Relation of active, passive, and quitting smoking with incident type 2 diabetes: a systematic review and meta-analysis	5	An Pan	Tangchun Wu	2015	CHN	The Lancet Diabetes and Endocrinology	596	59.6	Yes
Impact of Smokeless Tobacco Products on Cardiovascular Disease: Implications for Policy, Prevention, and Treatment: A Policy Statement From the American Heart Association	6	Mariann R. Piano	George Howard	2010	USA	Circulation	290	19.3	Yes

*Code for study type: 1: randomized controlled trial (RCT), primary report; 2: RCT, secondary analysis or follow-up; 3: cohort-based observational studies; 4: other observational studies; 5: meta-analysis; 6: Previous high-level (aggregated) evidence; 7: expert-based statements; 8: others, for example single-arm self-control studies

Table S3.1 continued from previous page

Title	Study type	First author	Last author	Year	Nationality	Journal	Cited by	Cited by per year	Sampled
Prospective, Cluster-Randomized Trial to Implement the Ottawa Model for Smoking Cessation in Diabetes Education Programs in Ontario, Canada	1	Robert D. Reid	Andrew L. Pipe	2018	CAN	Diabetes Care	26	3.7	Yes
Interventions With Adherence-Promoting Components in Pediatric Type 1 Diabetes: Meta-analysis of their impact on glycemic control	5	Korey K. Hood	Dennis Drotar	2010	USA	Diabetes Care	224	14.9	Yes
Applying Behavioral Economics Theories to Interventions for Persons with Diabetes	7	Susana R. Patton	Amy Hughes Lansing	2022	USA	Current Diabetes Reports	8	2.7	Yes
Cost-effectiveness of three doses of a behavioral intervention to prevent or delay type 2 diabetes in rural areas	2	Tiffany A. Radcliff	Michael G. Perri	2020	USA	Journal of the Academy of Nutrition and Dietetics	15	3	Yes
Evaluation of the Effects of 12 Weeks Mindfulness-Based Stress Reduction on Glycemic Control and Mental Health Indices in Women with Diabetes Mellitus Type 2	1	Omid Nikkhah Ravari	Anahita Babak	2020	IRA	Advanced Biomedical Research	18	3.6	Yes
Psychosocial outcomes in young adolescents with type 1 diabetes participating in shared medical appointments	1	Shideh Majidi	Jennifer K. Raymond	2021	USA	Pediatric Diabetes	14	3.5	Yes

*Code for study type: 1: randomized controlled trial (RCT), primary report; 2: RCT, secondary analysis or follow-up; 3: cohort-based observational studies; 4: other observational studies; 5: meta-analysis; 6: Previous high-level (aggregated) evidence; 7: expert-based statements; 8: others, for example single-arm self-control studies

Table S3.1 continued from previous page

Title	Study type	First author	Last author	Year	Nationality	Journal	Cited by	Cited by per year	Sampled
Effects of mindfulness- and acceptance-based interventions on diabetes distress and glycaemic level in people with type 2 diabetes: systematic review and meta-analysis.	5	Hau Yi Ngan	Wai Tong Chien	2021	HKG	DIABETIC MEDICINE	62	15.5	Yes
Kindness Matters: A Randomized Controlled Trial of a Mindful Self-Compassion Intervention Improves Depression, Distress, and HbA1c Among Patients With Diabetes	1	Anna M. Friis	Nathan S. Consedine	2016	NZL	Diabetes Care	301	33.4	Yes
Improving Quality Outcomes: The Value of Diabetes Care and Education Specialists	7	Kellie Rodriguez	Victor Phan	2022	USA	Clinical Diabetes	26	8.7	Yes
Related factors associated with fear of hypoglycemia in parents of children and adolescents with type 1 diabetes - A systematic review	6	Lu Zhang	Yu Zhang	2022	CHN	Journal of Pediatric Nursing	23	7.7	Yes
Effect of a collaborative care model on anxiety symptoms among patients with depression and diabetes in India: The INDEPENDENT randomized clinical trial	2	Christopher G. Kemp	Lydia A. Chwastiak	2022	IND	General Hospital Psychiatry	21	7	Yes
Cognitive behavior therapy for diabetes distress, depression, health anxiety, quality of life and treatment adherence among patients with type-II diabetes mellitus: a randomized control trial	1	Qasir Abbas	Washdev Washdev	2023	PAK	BMC Psychiatry	30	15	Yes

*Code for study type: 1: randomized controlled trial (RCT), primary report; 2: RCT, secondary analysis or follow-up; 3: cohort-based observational studies; 4: other observational studies; 5: meta-analysis; 6: Previous high-level (aggregated) evidence; 7: expert-based statements; 8: others, for example single-arm self-control studies

Table S3.1 continued from previous page

Title	Study type	First author	Last author	Year	Nationality	Journal	Cited by	Cited by per year	Sampled
Lifetime Duration of Depressive Disorders in Patients With Type 2 Diabetes	8	Mary de Groot	Jay H. Shubrook	2016	USA	Diabetes Care	68	7.6	Yes
The Pathways Study: A Randomized Trial of Collaborative Care in Patients With Diabetesand Depression	1	Wayne J. Katon	Terry Bush	2004	USA	JAMA Psychiatry	928	44.2	Yes
The Effect of Exercise Interventions to Improve Psychosocial Aspects and Glycemic Control in Type 2 Diabetic Patients: A Systematic Review and Meta-Analysis of Randomized Controlled Trials	5	Gholam Rasul Mohammad Rahimi	Vahid Saatchian	2021	IRA	Biological Research For Nursing	7	1.8	Yes
The association between type 2 diabetes and attention- deficit/hyperactivity disorder: A systematic review, meta-analysis, and population-based sibling study	5	Miguel Garcia-Argibay	Henrik Larsson	2023	SWE	Neuroscience and Biobehavioral Reviews	20	10	Yes
Severe Hypoglycemia and Cognitive Decline in Older People With Type 2 Diabetes: The Edinburgh Type 2 Diabetes Study	3	Insa Feinkohl	Jackie F. Price	2014	GBR	Diabetes Care	318	28.9	Yes
A Diabetes Mobile App With In-App Coaching From a Certified Diabetes Educator Reduces A1C for Individuals With Type 2 Diabetes	8	Shefali Kumar	Jessie L. Jusola	2018	USA	The Science of Diabetes Self-Management and Care	82	11.7	Yes
Digital Coaching Strategies to Facilitate Behavioral Change in Type 2 Diabetes: A Systematic Review	6	Bradley D Gershkowitz	Bradley H Crotty	2021	USA	The Journal of Clinical Endocrinology & Metabolism	32	8	Yes

*Code for study type: 1: randomized controlled trial (RCT), primary report; 2: RCT, secondary analysis or follow-up; 3: cohort-based observational studies; 4: other observational studies; 5: meta-analysis; 6: Previous high-level (aggregated) evidence; 7: expert-based statements; 8: others, for example single-arm self-control studies

Table S3.1 continued from previous page

Title	Study type	First author	Last author	Year	Nationality	Journal	Cited by	Cited by per year	Sampled
IMpact of flash glucose Monitoring in pEople with type 2 Diabetes Inadequately controlled with non-insulin Antihyperglycaemic ThErapy (IMMEDIATE): A randomized controlled trial	1	Ronnie Aronson	Ronald Goldenberg	2022	CAN	Diabetes, Obesity and Metabolism	48	16	Yes
Impact of Shift Work on Glycemic Control in Insulin Treated Diabetics Dar El Chefa Hospital	4	Ihab Mohammadi El Tayeb	Basma Kamal Ramadan	2014	EGY	International Journal of Diabetes Research	10	0.9	Yes
Sleep interventions and glucose metabolism: systematic review and meta-analysis	5	Vallari Kothari	Sirimon Reutrakul	2021	USA	Sleep Medicine	49	12.3	Yes
Learning in a Virtual Environment to Improve Type 2 Diabetes Outcomes: Randomized Controlled Trial	1	Constance M Johnson	Allison Vorderstrasse	2022	USA	JMIR Formative Research	8	2.7	Yes
The management of very low-calorie ketogenic diet in obesity outpatient clinic: a practical guide	7	Giovanna Muscogiuri, Luigi Barrea	Annamaria Colao	2019	ITA	Journal of Translational Medicine	163	27.2	Yes
The Evolution of Very-Low-Calorie Diets: An Update and Meta-analysis	5	Adam Gilden Tsai	Thomas A. Wadden	2012	USA	Obesity	538	41.4	Yes
Efficacy of dietary supplements containing isolated organic compounds for weight loss: a systematic review and meta-analysis of randomised placebo-controlled trials	5	Erica Bessell	Nicholas R. Fuller	2021	AUS	International Journal of Obesity	16	4	Yes

*Code for study type: 1: randomized controlled trial (RCT), primary report; 2: RCT, secondary analysis or follow-up; 3: cohort-based observational studies; 4: other observational studies; 5: meta-analysis; 6: Previous high-level (aggregated) evidence; 7: expert-based statements; 8: others, for example single-arm self-control studies

Table S3.1 continued from previous page

Title	Study type	First author	Last author	Year	Nationality	Journal	Cited by	Cited by per year	Sampled
Impact of weight loss on waist circumference and the components of the metabolic syndrome	8	Amy E Rothberg	William H Herman	2017	USA	BMJ Open Diabetes Research and Care	107	13.4	Yes
Reduction in the Incidence of Type 2 Diabetes with Lifestyle Intervention or Metformin	1	Diabetes Prevention Program Research Group	Diabetes Prevention Program Research Group	2002	USA	NEJM	25615	1113.7	Yes
2017 ACC/AHA/HFSA Focused Update of the 2013 ACCF/AHA Guideline for the Management of Heart Failure: A Report of the American College of Cardiology/American Heart Association Task Force on Clinical Practice Guidelines and the Heart Failure Society of America	6	Clyde W. Yancy	Cheryl Westlake	2017	USA	Circulation	5841	730.1	Yes
Cardiovascular Effects of Intensive Lifestyle Intervention in Type 2 Diabetes	1	The Look AHEAD Research Group	.	2013	USA	NEJM	3046	253.8	Yes
The efficacy and safety of meal replacement in patients with type 2 diabetes.	5	Wenjing Ye	Liehua Liu	2023	CHN	The Journal of Clinical Endocrinology & Metabolism	8	4	Yes
10-Year Follow-up of Intensive Glucose Control in Type 2 Diabetes	2	Rury R. Holman	H. Andrew W. Neil	2008	GBR	NEJM	10257	603.4	Yes

*Code for study type: 1: randomized controlled trial (RCT), primary report; 2: RCT, secondary analysis or follow-up; 3: cohort-based observational studies; 4: other observational studies; 5: meta-analysis; 6: Previous high-level (aggregated) evidence; 7: expert-based statements; 8: others, for example single-arm self-control studies

Table S3.1 continued from previous page

Title	Study type	First author	Last author	Year	Nationality	Journal	Cited by	Cited by per year	Sampled
Years of life gained by multifactorial intervention in patients with type 2 diabetes mellitus and microalbuminuria: 21 years follow-up on the Steno-2 randomised trial	2	Peter Gæde	Oluf Pedersen	2016	DNK	DIABETOLOGIA	1	0.1	Yes
Effect of early intensive multifactorial therapy on 5-year cardiovascular outcomes in individuals with type 2 diabetes detected by screening (ADDITION-Europe): a cluster-randomised trial	1	Simon J Griffin	Torsten Lauritzen	2011	GBR	The Lancet	569	40.6	Yes
Treatment intensification with stepwise addition of prandial insulin aspart boluses compared with full basal-bolus therapy (FullSTEP Study): a randomised, treat-to-target clinical trial	1	Helena W Rodbard	David HW Shu	2014	USA	The Lancet Diabetes and Endocrinology	118	10.7	Yes
The Effects of Dietary Education Interventions on Individuals with Type 2 Diabetes: A Systematic Review and Meta-Analysis	5	Juri Kim	Myung-Haeng Hur	2021	KOR	International Journal of Environmental Research and Public Health ...	24	6	Yes
Cardiorespiratory Fitness, BMI, Mortality, and Cardiovascular Disease in Adults with Overweight/Obesity and Type 2 Diabetes	2	WILLS, ANDREW C	KNOWLER, WILLIAM C.	2021	USA	Medicine & Science in Sports & Exercise	7	1.8	Yes

*Code for study type: 1: randomized controlled trial (RCT), primary report; 2: RCT, secondary analysis or follow-up; 3: cohort-based observational studies; 4: other observational studies; 5: meta-analysis; 6: Previous high-level (aggregated) evidence; 7: expert-based statements; 8: others, for example single-arm self-control studies

Table S3.1 continued from previous page

Title	Study type	First author	Last author	Year	Nationality	Journal	Cited by	Cited by per year	Sampled
Lifestyle Intervention and Medical Management With vs Without Roux-en-Y Gastric Bypass and Control of Hemoglobin A1c, LDL Cholesterol, and Systolic Blood Pressure at 5 Years in the Diabetes Surgery Study	2	Sayeed Ikramuddin	Charles J. Billington	2018	USA	JAMA	307	43.9	Yes
Long-term secondary prevention of cardiovascular disease with a Mediterranean diet and a low-fat diet (CORDIOPREV): a randomised controlled trial	1	Javier Delgado-Lista	Jose Lopez-Miranda	2022	ESP	The Lancet	316	105.3	Yes
Nutrition Therapy Recommendations for the Management of Adults With Diabetes	7	Alison B. Evert	William S. Yancy	2013	USA	Diabetes Care	2135	177.9	Yes
Salt Reduction to Prevent Hypertension and Cardiovascular Disease: JACC State-of-the-Art Review	5	Feng J. He	Graham A. MacGregor	2020	GBR	Journal of the American College of Cardiology	472	94.4	Yes
Association between Bout Duration of Physical Activity and Health: Systematic Review	6	John M Jakicic	Katrina L Piercy	2018	USA	Medicine & Science in Sports & Exercise	281	40.1	Yes
Are physical activity interventions for healthy inactive adults effective in promoting behavior change and maintenance, and which behavior change techniques are effective? A systematic review and meta-analysis	5	Neil Howlett	Angel Marie Chater	2018	GBR	Translational Behavioral Medicine	378	54	Yes

*Code for study type: 1: randomized controlled trial (RCT), primary report; 2: RCT, secondary analysis or follow-up; 3: cohort-based observational studies; 4: other observational studies; 5: meta-analysis; 6: Previous high-level (aggregated) evidence; 7: expert-based statements; 8: others, for example single-arm self-control studies

Table S3.1 continued from previous page

Title	Study type	First author	Last author	Year	Nationality	Journal	Cited by	Cited by per year	Sampled
Effectiveness of medical nutrition therapy in diabetes.	6	Pastors JG	Franz MJ	2012	USA	.	24	1.8	Yes
Effects of exercise on glycemic control and body mass in type 2 diabetes mellitus. A meta-analysis of controlled clinical trials.	5	Normand G. Boulé	Ronald J. Sigal	2001	CAN	JAMA	2984	124.3	Yes
Bariatric surgery: an IDF statement for obese Type 2 diabetes	7	J. B. Dixon	F. Rubino	2011	AUS	DIABETIC MEDICINE	953	68.1	Yes
Association between body mass index and mortality in an 80-year-old population	3	Yutaka Takata	Tadamichi Takehara	2007	JPN	JOURNAL OF THE AMERICAN GERIATRICS SOCIETY	92	5.1	Yes
Effect of Weight Loss and Exercise on Frailty in Obese Older Adults	1	Dennis T. Villareal	David R. Sinacore	2006	USA	JAMA Internal Medicine	389	20.5	Yes
Clinical Effectiveness of First and Repeat Influenza Vaccination in Adult and Elderly Diabetic Patients	4	Ingrid Looijmans-Van den Akker	Eelko Hak	2006	NLD	Diabetes Care	167	8.8	Yes
Brain Morphological Signatures for Chronic Pain	4	Marwan N. Baliki	A. Vania Apkarian	2011	USA	PLoS One	441	31.5	Yes
Pain Assessment and Management in Aging	7	Mimi Hanks-Bell	Judith A. Paice	2004	USA	Online Journal of Issues in Nursing	61	2.9	Yes

*Code for study type: 1: randomized controlled trial (RCT), primary report; 2: RCT, secondary analysis or follow-up; 3: cohort-based observational studies; 4: other observational studies; 5: meta-analysis; 6: Previous high-level (aggregated) evidence; 7: expert-based statements; 8: others, for example single-arm self-control studies

Table S3.1 continued from previous page

Title	Study type	First author	Last author	Year	Nationality	Journal	Cited by	Cited by per year	Sampled
Efficacy of treating pain to reduce behavioural disturbances in residents of nursing homes with dementia: cluster randomised clinical trial	1	Bettina S Husebo	Dag Aarsland	2011	NOR	BMJ	671	47.9	Yes
A randomized trial of intensive versus standard blood-pressure control	1	The SPRINT Research Group	The SPRINT Research Group	2015	USA	NEJM	6553	655.3	No
Effects of intensive blood-pressure control in type 2 diabetes mellitus	1	ACCORD Study Group	ACCORD Study Group	2010	USA	NEJM	4410	294	No
Intensive systolic blood pressure control and incident chronic kidney disease in people with and without diabetes mellitus: secondary analyses of two randomised controlled trials	2	Srinivasan Beddhu	Glenn M Chertow	2018	USA	The Lancet Diabetes and Endocrinology	110	15.71	No
Blood pressure targets for hypertension in people with diabetes mellitus	5	Jose Agustin Arguedas	James M Wright	2013	CRI	Cochrane Database of Systematic reviews	240	20	No
Blood pressure lowering for prevention of cardiovascular disease and death: a systematic review and meta-analysis	5	Dena Ettehad	Kazem Rahimi	2016	GBR	The Lancet	4200	466.67	No

*Code for study type: 1: randomized controlled trial (RCT), primary report; 2: RCT, secondary analysis or follow-up; 3: cohort-based observational studies; 4: other observational studies; 5: meta-analysis; 6: Previous high-level (aggregated) evidence; 7: expert-based statements; 8: others, for example single-arm self-control studies

Table S3.1 continued from previous page

Title	Study type	First author	Last author	Year	Nationality	Journal	Cited by	Cited by per year	Sampled
Effect of antihypertensive treatment at different blood pressure levels in patients with diabetes mellitus: systematic review and meta-analyses	5	Mattias Brunström	Bo Carlberg	2016	SWE	BMJ	509	56.56	No
Blood Pressure Targets in Subjects With Type 2 Diabetes Mellitus/Impaired Fasting Glucose: Observations From Traditional and Bayesian Random-Effects Meta-Analyses of Randomized Trials	5	Sripal Bangalore	Franz H. Messerli	2011	USA	Circulation	598	42.71	No
Effects of blood-pressure-lowering treatment on outcome incidence in hypertension 10 – Should blood pressure management differ in hypertensive patients with and without diabetes mellitus? Overview and meta-analyses of randomized trials	5	Thomopoulos, Costas	Zanchetti, Alberto	2017	GRC	Journal of Hypertension	304	38	No
Effects of intensive blood pressure lowering on cardiovascular and renal outcomes: updated systematic review and meta-analysis	5	Xinfang Xie	Anthony Rodgers	2016	CHN	The Lancet	1232	136.89	No
A Program to Prevent Functional Decline in Physically Frail, Elderly Persons Who Live at Home	1	Thomas M. Gill	Amy Byers	2002	USA	NEJM	1160	50.43	No
Intensive Weight Loss Intervention in Older Individuals: Results from the Action for Health in Diabetes Type 2 Diabetes Mellitus Trial	1	Mark A. Espeland	Helen P. Hazuda	2013	USA	Journal of the American Geriatrics Society	79	6.58	No

*Code for study type: 1: randomized controlled trial (RCT), primary report; 2: RCT, secondary analysis or follow-up; 3: cohort-based observational studies; 4: other observational studies; 5: meta-analysis; 6: Previous high-level (aggregated) evidence; 7: expert-based statements; 8: others, for example single-arm self-control studies

Table S3.1 continued from previous page

Title	Study type	First author	Last author	Year	Nationality	Journal	Cited by	Cited by per year	Sampled
Severe hypoglycemia and cognitive decline in older people with type 2 diabetes: the Edinburgh type 2 diabetes study	3	Insa Feinkohl	Jackie F. Price	2014	GBR	Diabetes Care	327	29.73	No
Severe hypoglycaemia, mild cognitive impairment, dementia and brain volumes in older adults with type 2 diabetes: the Atherosclerosis Risk in Communities (ARIC) cohort study	3	Alexandra K. Lee	Elizabeth Selvin	2018	USA	Diabetologia	103	14.71	No
Reduction in the incidence of type 2 diabetes with lifestyle intervention or metformin	1	William C Knowler	David M Nathan	2002	USA	NEJM	25978	1129.48	No
Cardiovascular mortality, all-cause mortality, and diabetes incidence after lifestyle intervention for people with impaired glucose tolerance in the Da Qing Diabetes Prevention Study: a 23-year follow-up study	2	Guangwei Li	Peter H Bennett	2014	CHN	The Lancet Diabetes and Endocrinology	808	73.45	No
Does diabetes prevention translate into reduced long-term vascular complications of diabetes?	7	David M. Nathan	Neil H. White	2019	USA	Diabetologia	85	14.17	No
The Cost-Effectiveness of Lifestyle Modification or Metformin in Preventing Type 2 Diabetes in Adults with Impaired Glucose Tolerance	2	William H. Herman	Robert E. Ratner	2005	USA	Annals of Internal Medicine	899	44.95	No

*Code for study type: 1: randomized controlled trial (RCT), primary report; 2: RCT, secondary analysis or follow-up; 3: cohort-based observational studies; 4: other observational studies; 5: meta-analysis; 6: Previous high-level (aggregated) evidence; 7: expert-based statements; 8: others, for example single-arm self-control studies

Table S3.1 continued from previous page

Title	Study type	First author	Last author	Year	Nationality	Journal	Cited by	Cited by per year	Sampled
The 10-year cost-effectiveness of lifestyle intervention or metformin for diabetes prevention: an intent-to-treat analysis of the DPP/DPPOS	2	The Diabetes Prevention Program Research Group	The Diabetes Prevention Program Research Group	2012	USA	Diabetes Care	534	41.08	No
Impact of the YMCA of the USA Diabetes Prevention Program on Medicare spending and utilization	4	Maria L. Alva	Lucia Rojas-Smith	2017	USA	Health Affairs	57	7.13	No
Cost-effectiveness of diabetes prevention interventions targeting high-risk individuals and whole populations: a systematic review	6	Xilin Zhou	Ping Zhang	2020	USA	Diabetes Care	151	30.2	No
Nutrition Therapy for Adults With Diabetes or Prediabetes: A Consensus Report	7			2019	USA	Diabetes Care	1418	236.33	No
Medical nutrition therapy and weight loss questions for the Evidence Analysis Library prevention of type 2 diabetes project: systematic reviews	6	Hollie A. Raynor	Lisa Moloney	2017	USA	Journal of the Academy of Nutrition and Dietetics	58	7.25	No
The effectiveness and cost of lifestyle interventions including nutrition education for diabetes prevention: a systematic review and meta-analysis.	5	Yu Sun	Brenda Davy	2017	USA	Journal of the Academy of Nutrition and Dietetics	239	29.88	No
Building therapeutic relationships: choosing words that put people first	7	Jane K. Dickinson	Melinda D. Maryniuk	2017	USA	Clinical Diabetes	41	5.13	No

*Code for study type: 1: randomized controlled trial (RCT), primary report; 2: RCT, secondary analysis or follow-up; 3: cohort-based observational studies; 4: other observational studies; 5: meta-analysis; 6: Previous high-level (aggregated) evidence; 7: expert-based statements; 8: others, for example single-arm self-control studies

Table S3.1 continued from previous page

Title	Study type	First author	Last author	Year	Nationality	Journal	Cited by	Cited by per year	Sampled
Effect of weight loss with lifestyle intervention on risk of diabetes	2	Richard F. Hamman	Judith Wylie-Rosett	2006	USA	Diabetes Care	1706	89.79	No
Exercise Training, Without Weight Loss, Increases Insulin Sensitivity and Postheparin Plasma Lipase Activity in Previously Sedentary Adults	8	Glen E. Duncan	Peter W. Stacpoole	2003	USA	Diabetes Care	649	29.5	No
Association of bariatric surgery with long-term remission of type 2 diabetes and with microvascular and macrovascular complications	3	Lars Sjöström	Lena M. S. Carlsson	2014	SWE	JAMA	1225	111.36	No
Dapagliflozin's Effects on Glycemia and Cardiovascular Risk Factors in High-Risk Patients With Type 2 Diabetes: A 24-Week, Multicenter, Randomized, Double-Blind, Placebo-Controlled Study With a 28-Week Extension	1	William T. Cefalu	Shamik J. Parikh	2015	USA	Diabetes Care	188	18.8	No
Food groups and risk of all-cause mortality: a systematic review and meta-analysis of prospective studies	5	Schwingshackl Lukas	Boeing Heiner	2017	AUT	The American Journal of Clinical Nutrition	659	82.38	No
An Update on the Mediterranean, Vegetarian, and DASH Eating Patterns in People With Type 2 Diabetes	7	Gretchen Benson	Joy Hayes	2020	USA	Diabetes Spectrum	26	5.2	No

*Code for study type: 1: randomized controlled trial (RCT), primary report; 2: RCT, secondary analysis or follow-up; 3: cohort-based observational studies; 4: other observational studies; 5: meta-analysis; 6: Previous high-level (aggregated) evidence; 7: expert-based statements; 8: others, for example single-arm self-control studies

Table S3.1 continued from previous page

Title	Study type	First author	Last author	Year	Nationality	Journal	Cited by	Cited by per year	Sampled
Efficacy and safety of carbohydrate counting versus other forms of dietary advice in patients with type 1 diabetes mellitus: a systematic review and meta-analysis of randomised clinical trials	5	Carlos E Builes-Montaño	Natalia A Rojas-Henao	2022	COL	Journal of Human Nutrition and Dietetics	26	8.67	No
Simplifying carb counting: A randomized controlled study – Feasibility and efficacy of an individualized, simple, patient-centred carb counting tool	1	Shulamit Witkow	Rachel Golan	2023	ISR	Endocrinology, Diabetes & Metabolism	9	4.5	No
A Randomized Crossover Trial to Compare Automated Insulin Delivery (the Artificial Pancreas) With Carbohydrate Counting or Simplified Qualitative Meal-Size Estimation in Type 1 Diabetes	1	Ahmad Haidar	Rémi Rabasa-Lhoret	2023	CAN	Diabetes Care	15	7.5	No
Prospective independent evaluation of the carbohydrate counting accuracy of two smartphone applications	8	Michael Joubert	Yves Reznik	2021	FRA	Diabetes Therapy	14	3.5	No
A Comparative Study on Carbohydrate Estimation: GoCARB vs. Dietitians	8	Maria F. Vasiloglou	Zeno Stanga	2018	CHE	Nutrients	86	12.29	No
The diabetes nutrition education study randomized controlled trial: A comparative effectiveness study of approaches to nutrition in diabetes self-management education	1	Michael E. Bowen	Russell L. Rothman	2016	USA	Patient Education and Counseling	137	15.22	No
Defining food literacy: A scoping review	7	Emily Truman	Charlene Elliott	2017	CAN	Appetite	358	44.75	No

*Code for study type: 1: randomized controlled trial (RCT), primary report; 2: RCT, secondary analysis or follow-up; 3: cohort-based observational studies; 4: other observational studies; 5: meta-analysis; 6: Previous high-level (aggregated) evidence; 7: expert-based statements; 8: others, for example single-arm self-control studies

Table S3.1 continued from previous page

Title	Study type	First author	Last author	Year	Nationality	Journal	Cited by	Cited by per year	Sampled
Effectiveness of Early Time-Restricted Eating for Weight Loss, Fat Loss, and Cardiometabolic Health in Adults With Obesity	1	Humaira Jamshed	Courtney M. Peterson	2022	USA	JAMA Internal Medicine	139	46.33	No
Effects of Time-Restricted Eating on Weight Loss and Other Metabolic Parameters in Women and Men With Overweight and Obesity	1	Dylan A. Lowe	Ethan J. Weiss	2020	USA	JAMA Internal Medicine	474	94.8	No
Nutrition Therapy Recommendations for the Management of Adults With Diabetes	7	Alison B. Evert	William S. Yancy, Jr.	2013	USA	Diabetes Care	2202	183.5	No
Review of current evidence and clinical recommendations on the effects of low-carbohydrate and very-low-carbohydrate (including ketogenic) diets for the management of body weight and other cardiometabolic risk factors: A scientific statement from the National Lipid Association Nutrition and Lifestyle Task Force	7	Carol F. Kirkpatrick	Kevin C. Maki	2019	USA	Journal of Clinical Lipidology	378	63	No
Efficacy and safety of low and very low carbohydrate diets for type 2 diabetes remission: systematic review and meta-analysis of published and unpublished randomized trial data	5	Joshua Z Goldenberg	Bradley C Johnston	2021	USA	BMJ	362	90.5	No

*Code for study type: 1: randomized controlled trial (RCT), primary report; 2: RCT, secondary analysis or follow-up; 3: cohort-based observational studies; 4: other observational studies; 5: meta-analysis; 6: Previous high-level (aggregated) evidence; 7: expert-based statements; 8: others, for example single-arm self-control studies

Table S3.1 continued from previous page

Title	Study type	First author	Last author	Year	Nationality	Journal	Cited by	Cited by per year	Sampled
Carbohydrate restriction for diabetes: rediscovering centuries-old wisdom	7	Belinda S. Lennerz	David S. Ludwig	2021	USA	The Journal of Clinical Investigation	60	15	No
A network meta-analysis on the comparative efficacy of different dietary approaches on glycaemic control in patients with type 2 diabetes mellitus	6	Lukas Schwingshackl	Heiner Boeing	2018	DEU	European Journal of Epidemiology	296	42.29	No
Academy of Nutrition and Dietetics Nutrition Practice Guideline for Type 1 and Type 2 Diabetes in Adults: Systematic Review of Evidence for Medical Nutrition Therapy Effectiveness and Recommendations for Integration into the Nutrition Care Process.	6	Marion J Franz	Megan Robinson	2017	USA	Journal of the Academy of Nutrition and Dietetics	380	47.5	No
Training in flexible, intensive insulin management to enable dietary freedom in people with type 1 diabetes: dose adjustment for normal eating (DAFNE) randomised controlled trial	1	DAFNE Study Group	DAFNE Study Group	2002	GBR	BMJ	1180	51.3	No
Diabetes Interactive Diary: A New Telemedicine System Enabling Flexible Diet and Insulin Therapy While Improving Quality of Life: An open-label, international, multicenter, randomized study	1	Maria C.E. Rossi	Giacomo Vespasiani	2010	ITA	Diabetes Care	232	15.47	No

*Code for study type: 1: randomized controlled trial (RCT), primary report; 2: RCT, secondary analysis or follow-up; 3: cohort-based observational studies; 4: other observational studies; 5: meta-analysis; 6: Previous high-level (aggregated) evidence; 7: expert-based statements; 8: others, for example single-arm self-control studies

Table S3.1 continued from previous page

Title	Study type	First author	Last author	Year	Nationality	Journal	Cited by	Cited by per year	Sampled
Glycaemic control and severe hypoglycaemia following training in flexible, intensive insulin therapy to enable dietary freedom in people with type 1 diabetes: a prospective implementation study	8	A. Sämann	U. A. Müller	2005	DEU	Diabetologia	267	13.35	No
Non?nutritive sweeteners for diabetes mellitus	5	Szimonetta Lohner	Joerg J Meerpohl	2020	HUN	Cochrane Database of Systematic reviews	87	17.4	No
2019 ACC/AHA Guideline on the Primary Prevention of Cardiovascular Disease: A Report of the American College of Cardiology/American Heart Association Task Force on Clinical Practice Guidelines	7	Donna K. Arnett	Boback Ziaeian	2019	USA	Journal of the American College of Cardiology	6272	1045.33	No
Nutrition therapy for adults with diabetes or prediabetes: a consensus report	7	Alison B Evert	William S Yancy Jr	2019	USA	Diabetes Care	1413	235.5	No
Consumption of Beverages Containing Low-Calorie Sweeteners, Diet, and Cardiometabolic Health in Youth With Type 2 Diabetes	2	Allison C. Sylvetsky	Laure El ghormli	2020	USA	Journal of the Academy of Nutrition and Dietetics	10	2	No
Exercise in adults with type 1 diabetes mellitus	7	Michael C. Riddell	Anne L. Peters	2022	CAN	Nature Reviews Endocrinology	51	17	No

*Code for study type: 1: randomized controlled trial (RCT), primary report; 2: RCT, secondary analysis or follow-up; 3: cohort-based observational studies; 4: other observational studies; 5: meta-analysis; 6: Previous high-level (aggregated) evidence; 7: expert-based statements; 8: others, for example single-arm self-control studies

Table S3.1 continued from previous page

Title	Study type	First author	Last author	Year	Nationality	Journal	Cited by	Cited by per year	Sampled
Lifestyle Interventions for Patients With and at Risk for Type 2 Diabetes: A Systematic Review and Meta-analysis	5	Elizabeth Sumamo Schellenberg	Christina Korownyk	2013	CAN	Annals of Internal Medicine	699	58.25	No
Evidence-based behavioral interventions to promote diabetes management in children, adolescents, and families.	7	Hilliard, Marisa E	Anderson, Barbara J.	2016	USA	American Psychologist	176	19.56	No
Effective strategies for encouraging behavior change in people with diabetes	7	Korey K Hood	Carolyn E Ievers-Landis	2015	USA	Diabetes Management	125	12.5	No
Effect of group cognitive behavioural therapy on psychological stress and blood glucose in people with type 2 diabetes mellitus: A community-based cluster randomized controlled trial in China	1	Chunrong Xu	Peian Lou	2020	CHN	Diabetic Medicine	28	5.6	No
Cognitive behavioral therapy in adolescents with type 1 diabetes: an integrative review	6	Kaitlyn Rechenberg	Rebecca Koerner	2021	USA	Journal of Pediatric Nursing	35	8.75	No
The efficacy of cognitive behavioral therapy-based intervention on patients with diabetes: A meta-analysis	5	Yanni Li	Jing Sun	2022	AUS	Diabetes Research and Clinical Practice	25	8.33	No

*Code for study type: 1: randomized controlled trial (RCT), primary report; 2: RCT, secondary analysis or follow-up; 3: cohort-based observational studies; 4: other observational studies; 5: meta-analysis; 6: Previous high-level (aggregated) evidence; 7: expert-based statements; 8: others, for example single-arm self-control studies

Table S3.1 continued from previous page

Title	Study type	First author	Last author	Year	Nationality	Journal	Cited by	Cited by per year	Sampled
Effectiveness of cognitive behavioral therapy-based interventions on psychological symptoms in adults with type 2 diabetes mellitus: An update review of randomized controlled trials	6	Eugenia Vlachou	Omar Cauli	2022	GRC	Journal of Diabetes and its Complications	33	11	No
Effect of a mindfulness programme for long-term care residents with type 2 diabetes: A cluster randomised controlled trial measuring outcomes of glycaemic control, relocation stress and depression	1	Shu-Ming Chen	Chiung-Jung (Jo) Wu	2020	TWN	International Journal of Older People Nursing	29	5.8	No
Evaluation of the Effects of 12 Weeks Mindfulness-Based Stress Reduction on Glycemic Control and Mental Health Indices in Women with Diabetes Mellitus Type 2	1	Nikkhah Ravari, Omid	Babak, Anahita	2020	IRA	Advanced Biomedical Research	23	4.6	No
Virtual group appointments reduce distress and improve care management in young adults with type 1 diabetes	1	Daniel I. Bisno	Jennifer K. Raymond	2022	USA	Journal of Diabetes Science and Technology	42	14	No
Effective interventions for reducing diabetes distress: systematic review and meta-analysis	5	Jackie Sturt	Lawrence Fisher	2015	GBR	International Diabetes Nursing	164	16.4	No
2022 National standards for diabetes self-management education and support	7	Jody Davis	Suzanne Villalobos	2022	USA	The Science of Diabetes Self-Management and Care	226	75.33	No

*Code for study type: 1: randomized controlled trial (RCT), primary report; 2: RCT, secondary analysis or follow-up; 3: cohort-based observational studies; 4: other observational studies; 5: meta-analysis; 6: Previous high-level (aggregated) evidence; 7: expert-based statements; 8: others, for example single-arm self-control studies

Table S3.1 continued from previous page

Title	Study type	First author	Last author	Year	Nationality	Journal	Cited by	Cited by per year	Sampled
Depression in Adults With Diabetes: Results of 5-yr Follow-Up Study	3	Patrick J Lustman	Ray E Clouse	1988	USA	Diabetes Care	393	10.62	No
Elevated Depression Symptoms, Antidepressant Medicine Use, and Risk of Developing Diabetes During the Diabetes Prevention Program	2	Richard R. Rubin	William C. Knowler	2008	USA	Diabetes Care	319	18.76	No
Effectiveness of Internet and Phone-Based Interventions on Diabetes Management of Children and Adolescents With Type 1 Diabetes: A Systematic Review	5	Xiaolei Zhao	Silin Zheng	2021	CHN	Worldviews on Evidence-based Nursing	10	2.5	No
Internet Delivered Diabetes Self-Management Education: A Review	6	Katherine Pereira	Allison Vorderstrasse	2015	USA	Diabetes Technology & Therapeutics	162	16.2	No
Long-Term Outcomes of a Web-Based Diabetes Prevention Program: 2-Year Results of a Single-Arm Longitudinal Study	8	S Cameron Sepah	Anne L Peters	2015	USA	Journal of Medical Internet Research	232	23.2	No
A Systematic Review of Reviews Evaluating Technology-Enabled Diabetes Self-Management Education and Support	6	Deborah A. Greenwood	Malinda Peeples	2017	USA	Journal of Diabetes Science and Technology	553	69.13	No
Long-Term Effects of a Novel Continuous Remote Care Intervention Including Nutritional Ketosis for the Management of Type 2 Diabetes: A 2-Year Non-randomized Clinical Trial	8	Shaminie J. Athinarayanan	James P. McCarter	2019	USA	Frontiers in Endocrinology	342	57	No

*Code for study type: 1: randomized controlled trial (RCT), primary report; 2: RCT, secondary analysis or follow-up; 3: cohort-based observational studies; 4: other observational studies; 5: meta-analysis; 6: Previous high-level (aggregated) evidence; 7: expert-based statements; 8: others, for example single-arm self-control studies

Table S3.1 continued from previous page

Title	Study type	First author	Last author	Year	Nationality	Journal	Cited by	Cited by per year	Sampled
Effectiveness and Safety of a Novel Care Model for the Management of Type 2 Diabetes at 1 Year: An Open-Label, Non-Randomized, Controlled Study	8	Sarah J. Hallberg	Jeff S. Volek	2018	USA	Diabetes Therapy	473	67.57	No
Telemedicine in the Management of Type 1 Diabetes	8	Timothy Xu	Mary Rhee	2018	USA	CDC Preventing Chronic Disease	161	23	No
Web-based interventions for dietary behavior in adults with type 2 diabetes: systematic review of randomized controlled trials	6	Jedha Dening	Ralph Maddison	2020	AUS	Journal of Medical Internet Research	47	9.4	No
Telehealth Interventions to Improve Diabetes Management Among Black and Hispanic Patients: a Systematic Review and Meta-Analysis	5	Andrew Anderson	Rishab Chimmanamada	2022	USA	Journal of Racial and Ethnic Health Disparities	50	16.67	No
Effect of Diabetes Health Coaching on Glycemic Control and Quality of Life in Adults Living With Type 2 Diabetes: A Community-Based, Randomized, Controlled Trial	1	Diana Sherifali	Hertzel C. Gerstein	2021	CAN	Canadian Journal of Diabetes	27	6.75	No
Telemedicine-Assisted Self-Management Program for Type 2 Diabetes Patients	1	Katja von Storch	Christiane Woopen	2019	DEU	Diabetes Technology & Therapeutics	113	18.83	No
Night-shift work is associated with poorer glycaemic control in patients with type 2 diabetes	3	Areesa Manodpitipong	Sirimon Reutrakul	2017	THA	Journal of Sleep Research	93	11.63	No

*Code for study type: 1: randomized controlled trial (RCT), primary report; 2: RCT, secondary analysis or follow-up; 3: cohort-based observational studies; 4: other observational studies; 5: meta-analysis; 6: Previous high-level (aggregated) evidence; 7: expert-based statements; 8: others, for example single-arm self-control studies

Table S3.1 continued from previous page

Title	Study type	First author	Last author	Year	Nationality	Journal	Cited by	Cited by per year	Sampled
Very-Low-Calorie Diets and Sustained Weight Loss	7	Wim H.M. Saris	Wim H.M. Saris	2012	NLD	Obesity Research	348	26.77	No
Micronutrient quality of weight-loss diets that focus on macronutrients: results from the A TO Z study	1	Gardner Christopher D	Cherin Rise	2010	USA	The American Journal of Clinical Nutrition	132	8.8	No
Effects of anti-obesity drugs, diet, and exercise on weight-loss maintenance after a very-low-calorie diet or low-calorie diet: a systematic review and meta-analysis of randomized controlled trials	5	Kari Johansson	Erik Hemmingsson	2014	SWE	The American Journal of Clinical Nutrition	338	30.73	No
UK prospective diabetes study 7: Response of fasting plasma glucose to diet therapy in newly presenting type II diabetic patients	1	UKPDS group	UKPDS group	1990	GBR	Metabolism	123	3.51	No
Beneficial health effects of modest weight loss	7	Goldstein DJ	Goldstein DJ	1992	USA	International Journal of Obesity and Related Metabolic Disorders	2066	62.61	No
The Evidence for the Effectiveness of Medical Nutrition Therapy in Diabetes Management	7	Joyce Green Pastors	Karmeen Kulkarni	2002	USA	Diabetes Care	728	31.65	No
Cardiovascular Effects of Intensive Lifestyle Intervention in Type 2 Diabetes	1	The Look AHEAD Research Group	The Look AHEAD Research Group	2013	USA	The NEJM	3139	261.58	No

*Code for study type: 1: randomized controlled trial (RCT), primary report; 2: RCT, secondary analysis or follow-up; 3: cohort-based observational studies; 4: other observational studies; 5: meta-analysis; 6: Previous high-level (aggregated) evidence; 7: expert-based statements; 8: others, for example single-arm self-control studies

Table S3.1 continued from previous page

Title	Study type	First author	Last author	Year	Nationality	Journal	Cited by	Cited by per year	Sampled
Eight-year weight losses with an intensive lifestyle intervention: The look AHEAD study	1	The Look AHEAD Research Group	The Look AHEAD Research Group	2013	USA	Obesity	851	70.92	No
Lifestyle Weight-Loss Intervention Outcomes in Overweight and Obese Adults with Type 2 Diabetes: A Systematic Review and Meta-Analysis of Randomized Clinical Trials	5	Marion J. Franz	Jeffrey J. VanWormer	2015	USA	Journal of the Academy of Nutrition and Dietetics	786	78.6	No
Long-term effects of intensive multifactorial therapy in individuals with screen-detected type 2 diabetes in primary care: 10-year follow-up of the ADDITION-Europe cluster-randomised trial	1	Simon J Griffin	Annelli Sandbæk	2019	GBR	The Lancet Diabetes & Endocrinology	51	8.5	No
Treatment intensification with stepwise addition of prandial insulin aspart boluses compared with full basal-bolus therapy (FullSTEP Study): a randomised, treat-to-target clinical trial	1	Helena W Rodbard	David HW Shu	2014	USA	The Lancet Diabetes & Endocrinology	122	11.09	No
Salt Reduction to Prevent Hypertension and Cardiovascular Disease: JACC State-of-the-Art Review	7	Feng J. He	Graham A. MacGregor	2020	GBR	Journal of the American College of Cardiology	530	106	No

*Code for study type: 1: randomized controlled trial (RCT), primary report; 2: RCT, secondary analysis or follow-up; 3: cohort-based observational studies; 4: other observational studies; 5: meta-analysis; 6: Previous high-level (aggregated) evidence; 7: expert-based statements; 8: others, for example single-arm self-control studies

Table S3.1 continued from previous page

Title	Study type	First author	Last author	Year	Nationality	Journal	Cited by	Cited by per year	Sampled
Physical activity, all-cause and cardiovascular mortality, and cardiovascular disease	6	William E Kraus	Katrina L Piercy	2019	USA	Medicine and science in sports and exercise	584	97.33	No
Behaviour change techniques targeting both diet and physical activity in type 2 diabetes: A systematic review and meta-analysis	5	Kevin A. Cradock	Kathleen A. Martin Ginis	2017	IRE	International Journal of Behavioral Nutrition and Physical Activity	383	47.88	No
Investigating weight loss in the elderly	7	GEORGE SZONYI	GEORGE SZONYI	2005	ZAF	Modern Medicine of South Africa	0	0	No

*Code for study type: 1: randomized controlled trial (RCT), primary report; 2: RCT, secondary analysis or follow-up; 3: cohort-based observational studies; 4: other observational studies; 5: meta-analysis; 6: Previous high-level (aggregated) evidence; 7: expert-based statements; 8: others, for example single-arm self-control studies

File S4

Causal linking words in guidelines and original studies

Table S4.1: Causal linking word frequencies

Guideline statement (n = 114)			OS abstract (n = 81)			OS main text (n = 183)		
Word	Wtd. Freq.	Unwtd. Freq.	Word	Wtd. Freq.	Unwtd. Freq.	Word	Wtd. Freq.	Unwtd. Freq.
benefit	14.3%	11.9%	associated	24.1%	21.1%	associated	17.5%	16.7%
reduce	12.5%	12.5%	reduce	15.4%	14.7%	effect	14.2%	13.1%
improve	11.7%	11.3%	effect	11.7%	10.5%	improve	13.0%	13.1%
associated	10.8%	9.52%	improve	7.82%	8.42%	reduce	10.3%	10.1%
effect	7.23%	5.95%	benefit	4.94%	4.21%	benefit	6.19%	7.07%
increase	6.29%	6.55%	lower	4.53%	5.26%	lower	3.01%	3.03%
maintain	2.19%	1.79%	greater	2.47%	3.16%	increase	2.73%	3.54%
help	2.05%	2.98%	promise<br>result in	2.47%	2.11%	difference	2.73%	2.53%
decrease	1.61%	1.79%	achieve<br>decrease	1.85%	2.11%	causal<br>cost effective<br>greater	2.19%	2.02%
risk	1.32%	1.79%	increase	1.65%	3.16%	lead	1.64%	2.02%
greater<br>link to<br>result in	1.32%	1.19%	better	1.65%	2.11%	help<br>promise<br>result in	1.64%	1.52%

Table S4.1 (Continued)

Guideline statement			OS abstract			OS main text		
Word	Wtd. Freq.	Unwtd. Freq.	Word	Wtd. Freq.	Unwtd. Freq.	Word	Wtd. Freq.	Unwtd. Freq.
contribute to	1.17%	1.19%	ameliorate	1.23%	1.05%	better	1.09%	1.01%
			consequence			inferior		
			contribute			superior		
			difference			useful		
			gain					
			higher					
			impact					
			more					
			significant					
			similar					
			superior					

OS, original study; Wtd., weighted; Freq., frequency; Unwtd., unweighted. The weighted and unweighted frequencies take the total number of occurrence of all causal linking words as denominator. For weighted frequency, each occurrence of word is weighted by the reciprocal of total numbers of causal linking words in the associated sentence. Words of same weighted and unweighted rankings are shown in one row. Word list is truncated at weighted frequency of 1.00% because there are too many ties to display with frequencies less than 1.00%. Confidence intervals and inference statistics for between-group comparison are not displayed, as the contents in this table serve for pure descriptive purpose.

References cited in supplementary files

1. Haber NA, Wieten SE, Rohrer JM, et al. Causal and Associational Language in Observational Health Research: A Systematic Evaluation. American Journal of Epidemiology 2022;191:2084–97.
2. Smit JM, Krijthe JH, Kant WMR, et al. Causal inference using observational intensive care unit data: a scoping review and recommendations for future practice. npj Digital Medicine 2023;6:1–11.
