# Supplementary material for: Causal language jumps and non-alignments between clinical practice guidelines and original studies: a systematic evaluation of diabetes guidelines and their cited evidence
Source: BMJ Open. 2026 Feb 5;16(2):e109205. doi: 10.1136/bmjopen-2025-109205 (PMC12878190; doi:10.1136/bmjopen-2025-109205)
Supplement: Supplementary file 2 [file bmjopen-2025-109205.reviewer_comments.pdf]

PEER REVIEW HISTORY

BMJ Open publishes all reviews undertaken for accepted manuscripts. Reviewers are asked to complete a checklist review form and are provided with free text boxes to elaborate on their assessment. These free text comments are reproduced below.

ARTICLE DETAILS

Title (Provisional)

Causal language jumps and non-alignments between clinical practice guidelines and original studies: a systematic evaluation of diabetes guidelines and their cited evidence

Authors

Wang , Keling; Wei, Chang; Labrecque, Jeremy

VERSION 1 - REVIEW

**Reviewer**	**1**
**Name**	**Xu, Huiwen**
**Affiliation**	**Yangzhou University**
**Date**	**25-Sep-2025**
**COI**	**None**

This manuscript addresses an important and often overlooked issue in evidence-based medicine: the misalignment between causal language used in clinical practice guidelines (CPGs) and the underlying cited evidence. The focus on diabetes guidelines provides a relevant and concrete context for exploring this "causal language jump." The topic is timely, methodologically sound in its systematic approach, and offers valuable insights for improving guideline development and critical appraisal. The paper is generally well-written and structured. However, several aspects require clarification and expansion to enhance the manuscript's rigor, reproducibility, and practical impact.

Methods：

The manuscript states: "we randomly drew 300 statements and picked their associated action recommendations..." "we randomly picked one original study for each guideline statement..."

While the use of random sampling is appropriate to mitigate selection bias, the exact method of randomization must be explicitly described to ensure reproducibility.

Adding a brief sentence detailing the randomization procedure is essential for methodological transparency.

Discussion ：

The discussion effectively summarizes the findings and their implications. However, it would be significantly enhanced by providing more concrete, actionable recommendations for guideline developers. Based on your results, consider suggesting specific steps they could take to minimize causal language jumps.

This would transform your valuable findings into a practical toolkit for improving future CPGs.

Other comments:

-In Table S1.2, the same word (e.g., "effect") appears in multiple categories ("Main text conclusive sentences" and "Main text action recommendations"). Please clarify the rationale behind this classification. A brief footnote or explanation in the table caption would resolve this potential confusion for the reader.

-For Table S3.1, the "Study type" column contains numbers (e.g., 1, 2, 3...) instead of descriptive text (e.g., "RCT," "Cohort study"). Please define what each number represents. This is most likely a key for study design types (e.g., 1 = Systematic Review, 2 = RCT, etc.). This key must be included either in the table itself, its caption, or in the supplementary materials' methods section.

-Regarding Table S4, please describe the operational definition or criteria used to classify a word as a "Causal linking word." Was it based on a pre-existing lexicon or linguistic theory? Was it defined solely by the authors? A sentence or two in the methods section explaining how these words were identified and selected would add clarity and methodological strength.

This is a thoughtful and well-conducted study that makes a novel contribution to the literature on evidence-based medicine and guideline development. The requested clarifications, particularly regarding methodology and definitions, are essential to uphold the paper's scientific rigor. The suggested expansion of the discussion would greatly increase its impact and utility for the target audience. I am optimistic that with these revisions, the manuscript will be suitable for publication.

**Reviewer**	**2**
**Name**	**Flory, James**
**Affiliation**	**Memorial Sloan Kettering Cancer Center**
**Date**	**28-Oct-2025**
**COI**	**None**

This interesting paper seeks to assess whether selected diabetes guideline make 'jumps' in asserting causality for their recommendations, beyond what is justified by underlying evidence.

Authors identified several major guidelines in diabetes management; selected eligible guideline recommendations and their surrounding statements; randomly sampled one of the surrounding statements; pulled the supporting studies for those sentences and pulled the most supportive concluding sentence. For all of these, the level of assertion of causality was rated by two raters using an established metric. The authors also checked the reporting quality of a randomly selected supporting study. This work resulted in a quantitative description of how often guideline recommendations exceed the causal confidence given by a supportive (aka surrounding) statement; how often surrounding statements exceeded the causal confidence given by their most strongly supporting reference; and how well reported those underlying references are. Overall it was found that jumps in causal confidence were common. The authors noted that this could mean that causal and associational language is not used optimally in guidelines or in the literature.

The work was challenging to follow. Some comments below about presenting more examples of assessment for causal jumps, more clearly, might help.

This paper has some significant strengths and some major weaknesses, which may be mainly a matter of presentation. Overall I recommend major revision.

Major strengths:

The biggest strength of the study is that the research question is both novel and important. An additional strength is the quantitative, structured approach used with its potential to provide objective measures of the quality of causal language in a guideline. An additional minor strength is that the project was pre-registered.

Major weaknesses:

The project has three major limitations.

First, it appears from the study description that association between study recommendations and a single supportive sentence are assessed and association between a surrounding sentence and a single reference are assessed. As the authors allude to in limitations, it could be the case that multiple references add up to stronger evidence than one by itself. This study was not designed to assess that kind of 'joint support'. It also seems possible that multiple surrounding sentences might all offer support to the same causal claim in a recommendation, and it appears that the methods used would miss that sort of 'joint support' as well. If this is correct this concern should be added to the limitations, and some empirical investigation done by the authors to see if this might be causing them to overestimate the frequency of causal jumps. This is the critical limitation of this manuscript and the one issue that if not thoroughly addressed might make it inappropriate to publish. In short, it's not going to be helpful to publish an assessment of causal links between

statements and 1:1 paired pieces of supporting evidence if such statements commonly rely on the joint support of several pieces of evidence.

Second, given the complexity and novelty of the method used, it would be helpful for the reader to be able to see the full context of the examples of causal jumps given. A key role this would play is in allowing the reader to reassure themselves that the structured methods employed are giving an accurate picture of the causal reasoning in the guidelines and that the 'joint support' problem is not severe. The paper's current structure makes this difficult if not impossible. For example, I tried to go to the original guideline and reference for example 3.1 in Table 3. I tried hard to find the underlying guideline and reference, but there did not seem to be any way for me to identify which guideline the recommendation came from, or the reference that was supposed to support the recommendations. It's essential to add references to this table and throughout the work so an interested reader can fully explore the context of the example. Expansion of table 3 to include more examples would also be helpful.

Third, there seems to be no objective referent against which to decide if the amount of causal jumps documented in the guidelines is excessive (in terms of frequency or size of the jump). Can the authors discuss?

VERSION 1 - AUTHOR RESPONSE

Reviewer 1
Dr. Huiwen Xu, Yangzhou University

Comment 1-1: This manuscript addresses an important and often overlooked issue in evidence- based medicine: the misalignment between causal language used in clinical practice guidelines (CPGs) and the underlying cited evidence. The focus on diabetes guidelines provides a relevant and concrete context for exploring this "causal language jump." The topic is timely, methodologically sound in its systematic approach, and offers valuable insights for improving guideline development and critical appraisal. The paper is generally well-written and structured. However, several aspects require clarification and expansion to enhance the manuscript's rigor, reproducibility, and practical impact.

Response 1-1: We thank the reviewer’s comments. We hope that the following changes and clarifications we made suffice to improve the readability and quality of our manuscript.

Comment 1-2: Methods: The manuscript states: "we randomly drew 300 statements and picked their associated action recommendations..." "we randomly picked one original study for each guideline statement..." While the use of random sampling is appropriate to mitigate selection bias, the exact method of randomization must be explicitly described to ensure reproducibility. Adding a brief sentence detailing the randomization procedure is essential for methodological transparency.

Response 1-2: We appreciate that the reviewer pointed this out. For the two places where we randomly selected items mentioned in this comment, we use function `sample` in base R 4.4 software to generate a sequence of randomly selected numbers with a fixed seed '114514'. This random sequence of length 300 (for guideline statements) or of length 1 (for one original study per statement) was then matched with the identifiers of the items, and the matched ones were those finally analyzed. Since we used a fixed seed number, this random sampling procedure should be reproducible. We have added detailed description of this sampling procedure right after the two sentences mentioned in this comment to clarify our procedure. Corresponding to this, we deleted one sentence about sampling in 'Data synthesis and statistical considerations' to avoid duplication.

Comment 1-3: Discussion: The discussion effectively summarizes the findings and their implications. However, it would be significantly enhanced by providing more concrete, actionable recommendations for guideline developers. Based on your results, consider suggesting specific steps they could take to minimize causal language jumps. This would transform your valuable findings into a practical toolkit for improving future CPGs.

Response 1-3: We thank the reviewer for giving this valuable suggestion. We have come up with several points that future guideline makers can think through if they wish. First, we suggest that there should always be researchers or practitioners with causal expertise joining the working group when making guidelines to collaborate with domain knowledge experts and provide synergy. Second, we suggest that even if a causal language jump, in its semantic sense, must be made (*e.g.* when an action recommendation must be actionable and provide detailed suggestions that come from domain expertise), such jump should be reported clearly and any decisions about such jump should be revealed transparently. Third, it is necessary for the guideline makers, together with causal inference researchers, to differentiate between "safe" and "dangerous" jumps. The safe ones should be reported as transparent as possible, while the dangerous ones should be eliminated. We have added such suggestions to the last paragraph of the discussion section, and we hope this provides future guideline makers with more insights and actionable toolkits.

Comment 1-4: Other comments: -In Table S1.2, the same word (e.g., "effect") appears in multiple categories ("Main text conclusive sentences" and "Main text action recommendations"). Please clarify the rationale behind this classification. A brief footnote or explanation in the table caption would resolve this potential confusion for the reader.

Response 1-4: We apologize for possible confusion caused by this table. In Table S1.2 (Table S2.2 in revised version), we aimed to display the most frequently used causal linking words in different sets of sentences in original studies that were evaluated in our study. There are four rows since we checked one conclusive sentence and one action recommendation sentence in both abstracts and main texts of the original studies, thus

forming in total four groups of sentences (see Methods section *'Original study selection and assessment'*). For each row, we listed the top 5 frequently used causal linking words in the corresponding group to illustrate how they are different in choosing and using these words. The numbers in the parentheses are the count of occurrence (appearance). We have added one paragraph before Table S2.2, a footnote following the table, and also modified the descriptions, and we wish these changes suffice to clarify the contents and aim of this table.

Comment 1-5: -For Table S3.1, the "Study type" column contains numbers (e.g., 1, 2, 3...) instead of descriptive text (e.g., "RCT," "Cohort study"). Please define what each number represents. This is most likely a key for study design types (e.g., 1 = Systematic Review, 2 = RCT, etc.). This key must be included either in the table itself, its caption, or in the supplementary materials' methods section.

Response 1-5: We apologize for the missing information in this table. Indeed we intended to use the numbers to represent different study types (1: RCT, primary report; 2: RCT, secondary analysis or follow-up; 3: cohort-based observational studies; 4: other observational studies; 5: meta-analysis; 6: Previous high-level (aggregated) evidence; 7: expert-based statements; 8: others, for example single-arm self-control studies) because the space for this table is limited. We have added this key list to the footnote of this table on each page, and added an asterisk mark in the table to notify the readers.

Comment 1-6: -Regarding Table S4, please describe the operational definition or criteria used to classify a word as a "Causal linking word." Was it based on a pre-existing lexicon or linguistic theory? Was it defined solely by the authors? A sentence or two in the methods section explaining how these words were identified and selected would add clarity and methodological strength.

Response 1-6: We take the formal definition of "causal linking words" as: the word that intend to express the existence, confidence, uncertainty, magnitude and other attributes of causality, and that plays a central role in causal language. Examples of such words include "effect", "benefit", "impact'", and "reduce"; these words express whether a (counterfactual) change exists and the direction of this change. This definition was based on a number of literature and previous common knowledge in the field of epidemiology and causal inference, and reference 3,4,5,7 in our manuscript supported or contributed to this definition.

Practically, we took a list of possible (in other words, candidate) causal linking words from Haber et al. (2022) paper (Ref 7 in manuscript; *Am J Epidemiol* 2022;191:2084-97. doi: 10.1093/aje/kwac137), in which they invited 47 independent reviewers to rate the causal implication of 'root words' extracted from top-journal published studies evaluating the relationship between a main exposure and an outcome (see Table 3 and 4 in this reference). This list is adopted in our study, and our rating procedure of the strength of causal language is also based on this list and the existing literature (ref 3 and

7 in manuscript).

We had this point in our first draft but decided to take it out as we wanted to keep the manuscript shorter. We have now added the related descriptions, definitions and examples of causal thinking words back, both in the introduction and the methods section. We hope these amendments suffice to explain how we define and select these causal linking words.

Comment 1-7: This is a thoughtful and well-conducted study that makes a novel contribution to the literature on evidence-based medicine and guideline development. The requested clarifications, particularly regarding methodology and definitions, are essential to uphold the paper's scientific rigor. The suggested expansion of the discussion would greatly increase its impact and utility for the target audience. I am optimistic that with these revisions, the manuscript will be suitable for publication.

Response 1-7: We appreciate that the reviewer gave many thorough and detailed comments about our work. We have accordingly addressed all the suggestions mentioned above, including adding more clarifications, checking definitions and adding one paragraph in discussion about future steps for guideline makers to look into causal language jumps and to think more causally. We hope that our revisions are clear and help with the improvement of the manuscript's quality towards publishing.

Reviewer: 2
Dr. J Flory, Memorial Sloan Kettering Cancer Center

Comment 2-1: This interesting paper seeks to assess whether selected diabetes guideline make 'jumps' in asserting causality for their recommendations, beyond what is justified by underlying evidence. Authors identified several major guidelines in diabetes management; selected eligible guideline recommendations and their surrounding statements; randomly sampled one of the surrounding statements; pulled the supporting studies for those sentences and pulled the most supportive concluding sentence. For all of these, the level of assertion of causality was rated by two raters using an established metric. The authors also checked the reporting quality of a randomly selected supporting study. This work resulted in a quantitative description of how often guideline recommendations exceed the causal confidence given by a supportive (aka surrounding) statement; how often surrounding statements exceeded the causal confidence given by their most strongly supporting reference; and how well reported those underlying references are. Overall it was found that jumps in causal confidence were common. The authors noted that this could mean that causal and associational language is not used optimally in guidelines or in the literature.
The work was challenging to follow. Some comments below about presenting more examples of assessment for causal jumps, more clearly, might help.
This paper has some significant strengths and some major weaknesses, which may be mainly a matter of presentation. Overall I recommend major revision.

Response 2-1: We express sincere gratitude to the reviewer for their thorough read our methodological article. The three weaknesses listed below are either not fully considered at the time of conducting this study, or not presented clearly when drafting the manuscript. In this reply, we give extensive explanations and discussion about each of the points mentioned below, and we have addressed them in our revised version of manuscript and also supplementary material. We hope these changes and explanations can potentially answer the reviewer's points.

Comment 2-2: Major strengths: The biggest strength of the study is that the research question is both novel and important. An additional strength is the quantitative, structured approach used with its potential to provide objective measures of the quality of causal language in a guideline. An additional minor strength is that the project was pre-registered.

Response 2-2: We thank the reviewer for this comment on the novelty and importance of our study.

Comment 2-3: Major weaknesses: The project has three major limitations.
First, it appears from the study description that association between study recommendations and a single supportive sentence are assessed and association between a surrounding sentence and a single reference are assessed. As the authors allude to in limitations, it could be the case that multiple references add up to stronger evidence than one by itself. This study was not designed to assess that kind of 'joint support'. It also seems possible that multiple surrounding sentences might all offer support to the same causal claim in a recommendation, and it appears that the methods used would miss that sort of 'joint support' as well. If this is correct this concern should be added to the limitations, and some empirical investigation done by the authors to see if this might be causing them to overestimate the frequency of causal jumps. This is the critical limitation of this manuscript and the one issue that if not thoroughly addressed might make it inappropriate to publish. In short, it's not going to be helpful to publish an assessment of causal links between statements and 1:1 paired pieces of supporting evidence if such statements commonly rely on the joint support of several pieces of evidence.

Note: there are two responses, 2-3-1 and 2-3-2, to this comment.

Response 2-3-1: We appreciate it very much that the reviewer pointed out this critical point of 'joint support' (that multiple evidence can be used to form arguments stronger than any of them to support a claim) between recommendation and guideline statements. Indeed, our methodology was not designed to assess this matter, and we ignored "recommendation to statement" but focused more on "statement to original study" when drafting the limitations of joint support in our manuscript. We fully understand the reviewer's concern, and we both dive into this issue here in the reply and in the article to try to reassure the reviewer and the reader's worry.

First, we provide a closer look at the action recommendation items with more than one guideline statement and also provide a breakdown of the number of causal language

jumps in Table R2-1. Most recommendations come with one or two guideline statements, and over two thirds of the observed causal language jumps are from those recommendations with less statements. Numerically speaking, we do not expect that the joint support problem possibly existing in the remaining 10 jumps would drive our main conclusion towards another direction. Please also note that the ascertainment of these jumps was through comparing the rating of a recommendation item and the highest rating among all the guideline statements that intend to support it. We added this table to our supplementary file (File S2.3, Table S2.1) and also added sentences in the Results section to give readers more information.

Table R2-1. Characteristics of action recommendations and causal language jumps (action recommendation vs. surrounding statements) by number of statements per recommendation

No. of guideline statements for one rec.	1	2	3	4	5	6	7	> 7
No. of rec.	27	18	14	9	5	2	4	4
Proportion in all rec. (N = 83)	32.5%	21.7%	16.9%	10.8%	6.02%	2.41%	4.82%	4.82%
No. of jumps observed according to our method	13	6	4	3	0	1	2	0
Proportion of jumps (N = 29)	44.8%	20.7%	13.8%	10.3%	0.00%	3.45%	6.90%	0.00%

Rec., action recommendation.

Second, we give here an example of the full context of the action recommendations with more than one statement in Table R2-2. It can be seen from this example that all the surrounding statements do not provide causal evidence or judgements for "why such screening at such frequency needs to be carried out", but only repeat that "it should be done" in different aspects. This example is perfectly in line with the fifth paragraph in our discussion section, that the guideline makers were trying to incorporate previous experience, expertise, and other subject- matter knowledge gained from practice and education to draft an actionable recommendation. It is clearly outlined here that, different sentences in a guideline often provide us with different aspects, among which the "joint support", or synergy, is less likely to exist. This example is added to our Table 4 (Examples) to provide our reader with more insights.

Table R2-2. Example of the full context of an recommendation supported by multiple statements

Action recommendations	Sampled and rated guideline statements	Full context
5.36 Diabetes care teams should implement psychosocial screening protocols for general and diabetes-related mood concerns as well as other topics such as stress, quality of life, available resources (financial, social, family, and emotional), and/or psychiatric history. Screening should occur at least annually	Topics to screen for may include, but are not limited to, attitudes about diabetes, expectations for treatment and outcomes (especially related to starting a new treatment or technology), general and diabetes-related mood, stress, and/or quality of life (e.g., diabetes distress, depressive symptoms, anxiety symptoms, and/or fear of hypoglycemia), available resources (financial, social, family, and emotional), and/or	Health care teams should develop and implement psychosocial screening protocols to ensure routine monitoring of psychosocial well-being and to identify potential concerns among people with diabetes, following published guidance and recommendations (406–411). *Topics to screen for may include, but are not limited to, attitudes about diabetes, expectations for treatment and outcomes (especially related to starting a new treatment or technology), general and diabetes-related mood, stress, and/or quality of life (e.g., diabetes distress, depressive symptoms, anxiety symptoms, and/or fear of hypoglycemia), available resources (financial, social, family, and emotional), and/or psychiatric history.* Given elevated rates of suicidality among people with diabetes (412–415), screening for suicidality may also be appropriate (416–418), similar to U.S. Preventive Services Task Force statements regarding screening for some adolescents and adults in the general population (419,420). A list of age-appropriate screening and evaluation measures is provided in the ADA position statement "Psychosocial Care for People with Diabetes" (1), and guidance has been published about selection of screening tools, clinical thresholds, and frequency of screening (408,421). Key opportunities for

or when there is a change in disease, treatment, or life circumstances. [ADA2024 Chr5 p. S91 rec. 5.36] Rating: *Moderate dependence*	psychiatric history. [ADA2024 Chr5 p. S92; col. 2 par. 2 sent. 2. Rating: *No relationship*]<br><br>Thus, screening for SDOH (e.g., loss of employment, birth of a child, or other family-based stresses) should also be incorporated into routine care (423). [ADA2024 Chr5 p. S92; col. 3 par. 1 sent. 2. Rating: *No relationship*]	psychosocial screening occur at diabetes diagnosis, during regularly scheduled management visits, during hospitalizations, with new onset of complications, during significant transitions in care such as from pediatric to adult care teams (422), at the time of medical treatment changes, or when problems with achieving A1C goals, quality of life, or self-management are identified. People with diabetes are likely to exhibit psychological vulnerability at diagnosis, when their medical status changes (e.g., end of the honeymoon period), when the need for intensified treatment is evident, and when complications are discovered. Significant changes in life circumstances and SDOH are known to considerably affect a person's ability to self-manage their condition. *Thus, screening for SDOH (e.g., loss of employment, birth of a child, or other family-based stresses) should also be incorporated into routine care (423).* In circumstances where individuals other than the person with diabetes are significantly involved in diabetes management (e.g., caregivers or family members), these issues should be monitored and treated by appropriate professionals (422,424,425).

We argue, further to this point, that although the 'joint support' problem exists in "recommendation-statement" and is of concern, it does not hinge us from making the argument that "causal language jump exists and needs to be either justified or re-considered". The "joint support" can even also be considered as a special type of 'discontinuity' because the guideline makers, or the person who decides on a stronger causal conclusion using the many pieces of evidence, should still explain transparently and explicitly the rationale behind such decision, or at least keep it in mind that. This is the same as the "safe jump" outlined in our second paragraph of discussion section. The assessment of contents and domain-specific knowledge that enable such synergy, is beyond not only the scope of this article, but also the capacity of average users and readers of a guideline. These 'joint support' should thus be reported and justified by guideline makers explicitly, as all other observed causal language jumps should be.

To summarize, we hope that the aforementioned several points, if possible, synthesize into a stronger "joint support" argument than any of them alone, to reassure the reviewer and our future readers that the mentioned issue exists but does not hinge the methodology we used from being valuable.

We have made the following amendments in our article and supplementary materials to reflect these points: (1) we have revised our limitations section to reflect this limitation and discussed its implication; (2) we have added one paragraph in discussion section (under "other considerations and suggestions") specifically about joint support problem and made arguments as mentioned above; (3) we added more examples for recommendations supported by multiple statements in our Example Table (Table 4, jump type 1b); (4) considering the length of our article, we added the Table R2-1 given above to supplementary file (File S2.3, Table S2.1) in case the reader wants to learn more, and we also revised the corresponding results section to provide more details; and (5) we also revised the "Strength and limitations" bullet points at the start of the article to reflect such changes.

Response 2-3-2: Beyond recommendation – statements joint support problem, we also outline here similarly about "joint support" in "statements – original studies". When checking guideline statement and original studies, "supported by multiple references" is not commonly observed, and causal language jumps in such statements are even rarer. Of

300 guideline statements that we sampled, 62% are not causal at all (in Table 2); among the remaining 114 causal ones, the number of references that were used to support them is as follows (Table R2-3):

Table R2-3. Characteristics of causal guideline statements and causal language jumps (original study to guideline statement) by number of reference following the statements

No. of reference in statements	0	0*	1	2	3	4	> 4
No. of statements	12	18	46	18	7	4	9
Proportion in causal statements (N = 114)	10.5%	15.8%	40.4%	15.8%	6.14%	3.51%	7.89%
Proportion in all statements (N = 300)	4.00%	6.00%	15.3%	6.00%	2.33%	1.33%	3.00%
No. of jumps observed, NOT accounting for alignment	12	3	13	3	0	0	0
Proportion of jumps observed (N = 31)	38.7%	9.68%	41.9%	16.7%	0.00%	0.00%	0.00%
No. of jumps observed, accounting for alignment	12	7	20	11	2	1	1
Proportion of jumps observed (N = 54)	22.2%	13.0%	37.0%	20.4%	3.70%	1.85%	1.85%

*: there is no reference following the statement itself, but a reference in its surrounding text is found to be related to this statement. See "methods: Original study selection and assessment" for details.

As outlined in the table above, there are 38/114 causal guideline statements with more than one reference, and it takes only 12% of all the 300 guidelines we sampled and checked. All the statements with >=3 reference are mostly effectively supported and there are very few jumps. To provide information for our readers on this matter, we have added details about the number of references under "Results – Alignment between original studies and guidelines" section, and we have put the table displayed below in our supplementary file under File S2.4 in case the reader wants to learn more details. We also provide examples under Table 4 for 'joint supported statements' (type 2c).

In summary, we think the possibility of 'joint support' is more a limitation that can happen to every methodology, than an incurable defect of our study. Future sophisticated methodological work relying on LLM-based rating and other natural language processing techniques would be promising to address this limitation. We have added several details in Results section, in supplementary files, and in Table 4 to try to provide more evidence for these arguments.

Comment 2-4: Second, given the complexity and novelty of the method used, it would be helpful for the reader to be able to see the full context of the examples of causal jumps given. A key role this would play is in allowing the reader to reassure themselves that the structured methods employed are giving an accurate picture of the causal reasoning in the guidelines and that the 'joint support' problem is not severe. The paper's current structure makes this difficult if not impossible. For example, I tried to go to the original guideline and reference for example 3.1 in Table 3. I tried hard to find the underlying guideline and

reference, but there did not seem to be any way for me to identify which guideline the recommendation came from, or the reference that was supposed to support the recommendations. It's essential to add references to this table and throughout the work so an interested reader can fully explore the context of the example.
Expansion of table 3 to include more examples would also be helpful.

Response 2-4: We fully agree that our Table 3 (Table 4 in the updated version) was not clear enough and it was hard to find the full context, and we did not consider this before. We have re- designed this table of examples. We added information (either an identifier + reference, or the page it appears + reference) for all the guideline statements, action recommendations, and original study sentences listed in Table 4 that allow the reader to find the source and context of them. We also expanded this table to include more examples to give the reader a better sense what a jump may look like.

Regarding a closer look of 'jumps when joint support is suspicious', we also included two additional type of jump (type 1b: causal language jump between recommendation items and multiple guideline statements, as mentioned in response 2-3; and type 2c: causal language jump between guideline statements that are supported by multiple original studies" and picked up a number of examples. For other changes, analyses and explanations that try to reassure the reader, we have elaborated them in Response 2-3-1 and 2-3-2.

Regarding the current structure of the paper that may make it difficult to follow, we have revised our discussion section where we extensively discussed the results and their interpretation. We added subtitles to this long discussion section and outlined the several critical points we want to make using these subtitles. Details of guidelines are also added to the Result section. We have to apologize that this is partly due to the novel question we answer and the novel method we use.
We hope that after the figures, especially Figure 1 that explain a lot about the situation of this study come into their place and are displayed in text, the readability of our paper will be further improved.

Comment 2-5: Third, there seems to be no objective referent against which to decide if the number of causal jumps documented in the guidelines is excessive (in terms of frequency or size of the jump). Can the authors discuss?

Response 2-5: We thank the reviewer for pointing this critical point out. The reason why the article does not provide objective standard or criterion about "how many causal language jumps are too many" is threefold.

First, our article is the first to evaluate the causal language jumps within the context of clinical practice guidelines, therefore it is hard to find pre-existing references or criteria to the frequency/size of the jump in this specific context. In one of our key reference articles (Haber et al. 2022; Ref 7 in manuscript, doi: 10.1093/aje/kwac137) from which we also took our rating scales and causal linking words, it was reported that "*Of the 34% (n = 400) of studies with action recommendations, 15.3% had action recommendations*

that implied causality less strongly than the linking sentence, 40.3% were commensurate, and 44.5% were stronger (Figure 3A)". This is the only numerical value that could be used to compare with our findings. However, the Haber 2022 article considers a "jump" within a single study and only for observational settings, which deviates substantially from our context. Therefore, we think it would be good to outline this (which is stated in Discussion section, see the fifth paragraph) but would not be useful to compare or to judge if the number of jumps found in our study is too high. If future studies are willing to use our methodological framework, our results could be a reference to them.

Second, as outlined in our discussion section, we wanted to reiterate that causal language jumps do not necessarily come with "something wrong", and that causal language jumps are heterogeneous, which means there should not be a consistent, objective criteria determining how many is "too many". A causal language jump can be the result of many possibilities; their commonality is that the semantic discontinuity of causality happens somewhere, and justifications should be explicitly made. This discontinuity can be either naturally justified, or justified by the person who makes such jump, or due to the messy use of causal linking words and the many unspoken rules in academic publishing, or from misinterpretation of causal evidence. Some are more acceptable than others, and for different users (clinicians vs. caregivers vs. researchers) the interpretation also differs. On semantic or on methodological level, without the help of domain experts, one cannot, and should not differentiate between these situations easily, let alone giving a criterion for the frequency of the jumps.

Last, if we were to differentiate the jumps detected in our study into "safe" and "dangerous" ones, we would have come up with the following criteria: (1) there is no limit for safe jumps, provided that all of them are necessary, and have been transparently reported with the help of causal inference researchers; and (2) there should not be any dangerous jumps. However, as discussed, it is not possible nor necessary for this methodological paper to judge over which jumps are safe or not. Guideline developers should keep this in mind and should work hard towards this aim.

We apologize that we did not fully clarify this point in our discussion and spread it over several paragraphs. We have added such discussion specifically about the criterion of number of jumps to the second paragraph of the discussion section. In the last paragraph before limitations, we discussed "the ideal status". We hope this gives our readers a clearer picture of how to interpret the results.

VERSION 2 - REVIEW

**Reviewer**	**2**
**Name**	**Flory, James**
**Affiliation**	**Memorial Sloan Kettering Cancer Center**
**Date**	**16-Dec-2025**

COI

Very thorough and thoughtful revision.
